# Adaptations in equine appendicular muscle activity and movement occur during induced fore- and hindlimb lameness: An electromyographic and kinematic evaluation

**DOI:** 10.3389/fvets.2022.989522

**Published:** 2022-11-08

**Authors:** Lindsay B. St. George, Tijn J. P. Spoormakers, Ineke H. Smit, Sarah Jane Hobbs, Hilary M. Clayton, Serge H. Roy, Paul René van Weeren, Jim Richards, Filipe M. Serra Bragança

**Affiliations:** ^1^Research Centre for Applied Sport, Physical Activity and Performance, University of Central Lancashire, Preston, United Kingdom; ^2^Section Equine, Department of Clinical Sciences, Faculty of Veterinary Medicine, Utrecht University, Utrecht, Netherlands; ^3^Department of Large Animal Clinical Sciences, College of Veterinary Medicine, Michigan State University, East Lansing, MI, United States; ^4^Delsys/Altec Inc., Natick, MA, United States; ^5^Allied Health Research Unit, University of Central Lancashire, Preston, United Kingdom

**Keywords:** horse, surface electromyography, sEMG, gait analysis, biomechanics, limb movement, forelimb, trot

## Abstract

The relationship between lameness-related adaptations in equine appendicular motion and muscle activation is poorly understood and has not been studied objectively. The aim of this study was to compare muscle activity of selected fore- and hindlimb muscles, and movement of the joints they act on, between baseline and induced forelimb (iFL) and hindlimb (iHL) lameness. Three-dimensional kinematic data and surface electromyography (sEMG) data from the fore- (triceps brachii, latissimus dorsi) and hindlimbs (superficial gluteal, biceps femoris, semitendinosus) were bilaterally and synchronously collected from clinically non-lame horses (*n* = 8) trotting over-ground (baseline). Data collections were repeated during iFL and iHL conditions (2–3/5 AAEP), induced on separate days using a modified horseshoe. Motion asymmetry parameters and continuous joint and pro-retraction angles for each limb were calculated from kinematic data. Normalized average rectified value (ARV) and muscle activation onset, offset and activity duration were calculated from sEMG signals. Mixed model analysis and statistical parametric mapping, respectively, compared discrete and continuous variables between conditions (α= 0.05). Asymmetry parameters reflected the degree of iFL and iHL. Increased ARV occurred across muscles following iFL and iHL, except non-lame side forelimb muscles that significantly decreased following iFL. Significant, limb-specific changes in sEMG ARV, and activation timings reflected changes in joint angles and phasic shifts of the limb movement cycle following iFL and iHL. Muscular adaptations during iFL and iHL are detectable using sEMG and primarily involve increased bilateral activity and phasic activation shifts that reflect known compensatory movement patterns for reducing weightbearing on the lame limb. With further research and development, sEMG may provide a valuable diagnostic aid for quantifying the underlying neuromuscular adaptations to equine lameness, which are undetectable through human observation alone.

## Introduction

Pain reduction during lameness is mainly achieved by redistributing load from the painful limb onto the non-lame or less lame limbs, which is manifested as compensatory movement (1–7) and is thought to be perpetuated by altered (adaptive) muscle activity (8). Equine veterinarians spend the majority of their working time on lameness consultation (9), which is the most common cause of occupational disability in horses (10, 11) and reason for euthanasia and/or other forms of wastage in geriatric (12) and ridden horses (10, 11, 13). Despite the high prevalence and the large impact of lameness on equine welfare and performance, as well as the financial (14) and emotional (15, 16) burden placed on horse owners, veterinary lameness evaluation remains heavily reliant on subjective assessment of asymmetries in head movement, or “head nod,” and asymmetries in pelvic movement, or “hip hike,” as the main indicators of fore- and hindlimb lameness, respectively (3, 17). More recently, advancements in equine gait analysis have provided veterinarians with objective lameness detection methods that are increasingly employed to assist in clinical decision-making (18, 19). Still, the main criteria for visual and objective lameness assessment are the presence and degree of global changes/asymmetries in the vertical height of the head and pelvis (18, 19). These are induced by the gait abnormalities we recognize as lameness and are assumed to be effectuated through adaptations in muscle contraction and coordination. However, the neuromuscular mechanisms are poorly understood, mainly because diagnostic methods have not been synchronized with gait analysis systems to accurately quantify isolated muscle function and/or underlying neuromuscular adaptations to lameness. Knowledge of adaptive muscle activity during equine lameness is a missing link in understanding the etiology and clinical signs of lameness and research is required to fill this gap in knowledge.

Surface electromyography (sEMG) represents an excellent tool for exploring these unanswered questions about equine neuromuscular control. It offers a non-invasive, quantitative means of estimating the degree of muscle activation by recording a summation of motor unit action potentials from electrodes placed on selected superficial muscles (20). sEMG has been successfully used in human research to investigate compensatory neuromuscular strategies for various musculoskeletal and neurological disorders such as low back pain (21), Parkinson's disease (22) stroke (23), spinal cord injury (24) and fibromyalgia (25). Following decades of research in the human field, sEMG is recommended by the American Academy of Neurology as a clinical tool for the kinesiologic analysis of movement disorders (26). In humans, sEMG can be used to classify movement disorders based on the amplitude and phasic activity patterns of muscle activation (21–27). In the veterinary field, sEMG has been identified as a potential diagnostic method for evaluating normal and dysfunctional muscle activity (28), but thus far research establishing its clinical utility in horses is lacking. In the dog, sEMG has been used to study compensatory neuromuscular strategies in animals with induced unilateral hindlimb lameness (29) and hip osteoarthritis (30) with both studies reporting significant alterations in the amplitude and timing of muscular activation.

sEMG is gaining popularity in equine biomechanics research mainly due to significant advancements in sensor technology, custom wireless transmission protocols, and electromechanically stable interfaces. Collectively, these advances make it possible for researchers to acquire high fidelity sEMG signals from a large animal in motion. Most of the studies to date have investigated muscle activity of selected superficial muscles during normal locomotion (31–34), and to the authors best knowledge, only two known studies have investigated the relationship between lameness and neuromuscular function in horses (8, 35). In comparison to a group of non-lame horses, Zaneb et al. (8) reported significantly lower sEMG amplitude ratios from semitendinosus and gluteus medius muscles of the non-lame limb and lame limb, respectively, in lame horses during treadmill trot. The authors interpreted this adaptation as a “more distinct resting phase” between active contractions of gluteus medius and semitendinosus in lame horses at trot (8). However, kinematics were not considered by Zaneb et al. (8) and the use of chronic lameness cases, where the degree and location of lameness were not standardized, makes between- and within-group comparisons questionable and interpretation toward causal relationships even more daunting. In another study, King et al. (35) measured activity of selected thoracic limb muscles using fine-wire and surface electromyography, alongside ground reaction forces and kinematics, in horses with induced, unilateral carpal joint osteoarthritis during overground trot. sEMG signals revealed significantly delayed activation of the ulnaris lateralis within the lame forelimb, which was interpreted as muscular weakness or dysfunction relating to stabilization of the carpal joint during stance (35). Although these studies provide preliminary insight into equine muscle function during lameness, further research is required to comprehensively quantify muscular adaptations within the thoracic and pelvic limbs during both fore- and hindlimb lameness conditions.

The induction of standardized, mild and temporary lameness, allows the researcher to investigate compensatory mechanisms in animals with a known diagnosis and minimal variation across subjects (18, 36). This is an essential consideration for preliminary research in this field. More importantly, research has yet to combine sEMG with existing gait kinematic asymmetry parameters to explore the critical relationship between adaptive movement and muscle activity during lameness. Thus, the aim of this study was to compare muscle activity of selected fore- and hindlimb muscles, and movement of the joints they act on, between baseline and induced forelimb (iFL) and hindlimb (iHL) lameness. We hypothesize that adaptations in muscle activity occur during specific phases of gait under iFL and iHL conditions. We also hypothesize that these muscular adaptations are identifiable using sEMG and result in the altered kinematics that are characteristic of the lameness in individual horses when compared to their non-lame condition.

## Materials and methods

Ethical approval for this study was obtained from Utrecht University (CCD: AVD108002015307) and the University of Central Lancashire (Reference number: RE/17/08a_b).

### Horses

Eight (n = 8) horses (sex: 7 mares, 1 stallion, age: 9.2 ± 3.9 years, height: 161.3 ± 3.4 cm, body mass: 582.1 ± 39.4 kg, breed: 7 Dutch Warmblood, 1 Friesian) were used. Horses were part of the university herd, were in regular use for low-level dressage and pleasure riding and were accustomed to being walked and trotted in-hand. Horses were deemed as clinically non-lame (<1/5 AAEP Lameness Scale) through visual assessments by two qualified veterinarians (T. S., F. S. B).

### Instrumentation and equipment set up

#### Surface electromyography (sEMG)

**Figure 1:**
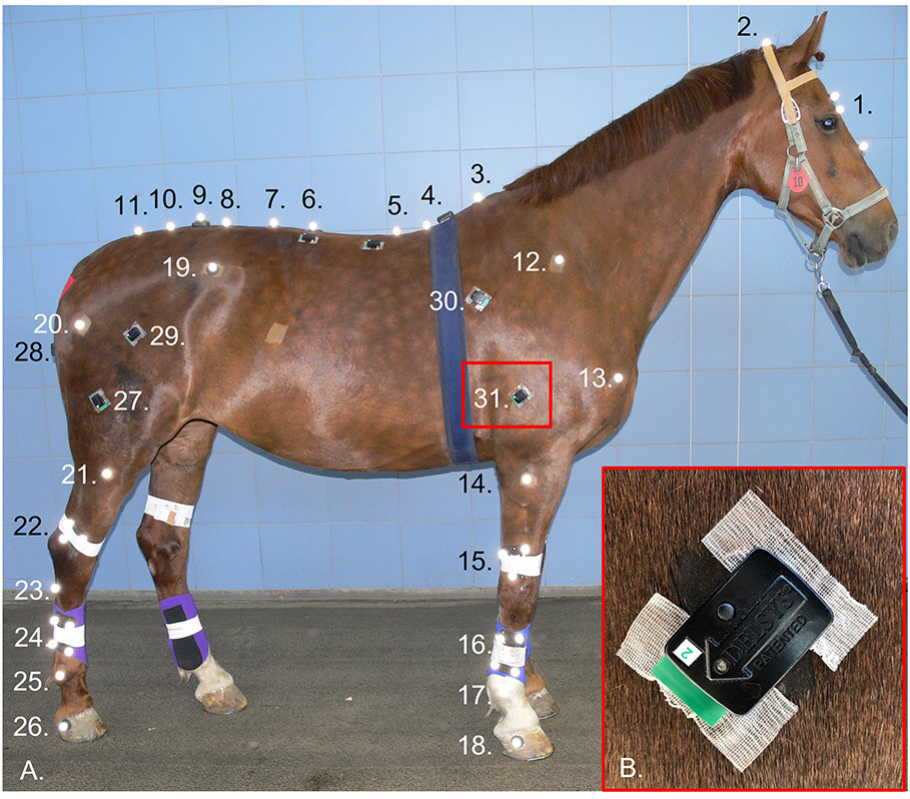
Retro-reflective markers and surface electromyography (sEMG) sensors attached to one subject **(A)** at the following anatomical locations: 1. Marker cluster attached to head, 2. Poll, 3. T6, 4. T10, 5. T13, 6. L1, 7. L3, 8. L5, 9. Between the tubera sacrale, 10. S3, 11. S5, and bilaterally over: 12. Proximal end spina scapulae, 13. Greater tubercle of the humerus, 14. Lateral tuberosity radius, 15. Marker cluster attached to distal radius, 16. Marker cluster attached to mid 3rd metacarpal bone, 17. Center of rotation metacarpophalangeal joint, 18. Lateral hoof wall (approximately center of rotation of the distal interphalangeal joint, 19. Tuber coxae, 20. Greater trochanter of the femur, 21. Lateral tibia plateau, 22. Marker cluster attached to distal tibia, 23. Proximal end 4th metatarsal bone, 24. Marker cluster attached to mid 3rd metatarsal bone, 25. Center of rotation metatarsophalangeal joint, 26. Lateral hoof wall. Bilateral sEMG sensor sites for 27. Biceps femoris, 28. Semitendinosus, 29. Superficial gluteal, 30. Latissimus dorsi, 31. Triceps brachii. Inset **(B)**: showing prepared skin site and adhesion technique for sEMG sensors.

sEMG sensors were positioned to record bilaterally from the following superficial appendicular muscles: long head of triceps brachii (triceps), latissimus dorsi (latissimus), superficial gluteal (gluteal), vertebral head of biceps femoris (biceps) and semitendinosus. These muscles were chosen as they are “prime movers” and thus purported to exhibit adaptive functionality during lameness. Wireless sEMG sensors (Delsys Trigno, Delsys Inc., USA), with a bipolar parallel bar electrode configuration and a fixed interelectrode distance of 10 mm, were employed. A combination of ultrasonography and previously described anatomical locations and/or sEMG sensor locations (37–40) were used to accurately determine sensor sites above each muscle. Sensor sites are illustrated in Figure 1 and were located as follows, triceps: approximately midway along and ~5 cm cranial to a line joining the olecranon and proximal point of the scapular spine (37, 38), biceps: approximately midway between greater trochanter and patella, 12–18 cm cephalad to the cranial margin of semitendinosus (38, 39), semitendinosus: midway between tuber ischii and caudal surface of femorotibial joint (40). Given the lack of published descriptions for sEMG sensor sites over latissimus and superficial gluteal muscles, sites for these muscles were primarily determined using ultrasonography as follows: latissimus: approximately midway along the scapula and positioned caudally from this point on a line that vertically intersects the withers at approximately T8–T9, gluteal: cranial to the greater trochanter at a point approximately midway along a line drawn between the tubera sacrale and greater trochanter (Figure 1).

After the sensor sites were determined, the overlying hair was removed using clippers (No. 40 clipper blade), and the skin was thoroughly cleaned using isopropyl alcohol. A small amount of saline solution was applied to each electrode bar to act as an electrolytic solution (41, 42). Sensors were then positioned on the muscle belly, with the electrodes oriented perpendicular to the underlying muscle fiber direction (43, 44), as determined using ultrasonography, and attached to the skin using Delsys Adhesive Surface Interface strips (Delsys Inc., USA). For additional adhesion, a drop of cyanoacrylate glue was placed on top of double-sided tape attached to the top and bottom of the sensor, above each electrode pair (Figure 1).

#### Kinematics

Three-dimensional (3D) kinematic data were collected using an optical motion capture system of 18 high-speed, infrared cameras (Oqus 700+, Qualisys AB, Sweden), which were secured to the walls of a large indoor hall, where veterinary lameness examinations are conducted. The system was calibrated for each data collection session, producing a large calibration volume ~56 m long and 10 m wide. The optical motion capture system was hardware synchronized to the sEMG system to record the two sets of time series data in one file for further processing. To collect 3D kinematic data, retro-reflective markers (19 mm diameter, super-spherical markers, Qualisys AB, Sweden) were positioned over anatomical landmarks (Figure 1). Hair was clipped from the marker locations to ensure optimal adhesion and consistent placement across data collection sessions. Markers were attached using double-sided adhesive tape, with an additional drop of cyanoacrylate glue used to secure the hoof and distal limb markers. Marker clusters were applied to the head with double-sided tape and bandaged onto the third metacarpus, third metatarsus, radius, and tibia (Figure 1).

### Data acquisition protocol

To simulate a real-world lameness examination, sEMG (2,000 Hz) and 3D kinematic (200 Hz) data were synchronously collected from in-hand trot trials, conducted on a straight, hard-surfaced, indoor runway during control (baseline 1, baseline 2) and induced lameness (iFL, iHL) conditions. Data were collected using Qualisys Track Manager (Qualisys AB, Sweden) software. Each horse undertook four passes of the straight runway and horses were permitted to trot at their preferred velocity. One handler led the horses during in-hand trot trials. After collecting data for the control (baseline 1) condition, temporary, mild iFL (2–3/5 AAEP Lameness Scale) was induced by qualified veterinarians (T. S., F. S. B.) using a mechanical, flat headed screw to exert pressure on the sole of the hoof using a modified horseshoe (36). The veterinarians graded and monitored the resulting lameness.

Horses were randomly divided into two groups (*n* = 4) for right and left iFL, in a cross-over design. Following iFL, trot trials were repeated. After a washout period of at least 24 h, the same data collection process was repeated for control (baseline 2) and iHL conditions, where iHL was again randomized to the right (*n* = 4) or left (*n* = 4) HL. After each data collection session, the screw/sole pressure was removed. None of the horses showed adverse reactions to the mild, temporary lameness that was induced for the purposes of this study.

### Data analysis

#### sEMG signal processing and analysis

Raw sEMG signals were differentially amplified by a factor gain of 909, a common-mode rejection ratio of >80 dB and an internal Butterworth high-pass (20 ± 5 Hz cut-off, >40 dB/dec) and low-pass filter (450 ± 50 Hz cut-off, >80 dB/dec). Post-processing and analysis of sEMG signals was conducted in Visual3D (Version 2021.06.2, C-Motion Inc., USA). Post-processing of sEMG signals included DC-offset removal, followed by the application of a high-pass filter (Butterworth 4th order, 40 Hz cut-off) to attenuate low-frequency noise contamination (45), and then full-wave rectification. Discrete sEMG variables included average rectified value (ARV) and timings of sEMG activity onset, offset, and resultant activity duration for each muscle across all strides.

ARV was calculated using full-wave rectified signals with stride duration as the temporal domain. As left/right sides of each muscle were analyzed separately, contralateral hindlimb impact events were employed for stride segmentation of sEMG signals. Outliers in ARV data were detected and removed by setting upper and lower outlier limits as two standard deviations outside of the mean ARV values within each horse, muscle, and condition (baseline 1, baseline 2, iFL, iHL) (38). Within-horse ARV data were normalized to a reference voluntary contraction (RVC): the maximum value observed for each muscle across all strides from the corresponding baseline condition (46). This permitted examination of the proportional change in muscle activity between baseline 1 and iFL and baseline 2 and iHL conditions.

Muscle activity onset and offset events were calculated across strides, in accordance with the double threshold method, described by St. George et al. (38). Events were calculated from enveloped signals (Butterworth 4th order, low-pass filter, 10 Hz cut-off), with an amplitude threshold defined as 10% of the peak amplitude value of each individual sEMG signal and the timing threshold defined as 5% of the average gait cycle duration from the control condition across all horses (38). In accordance with St. George et al. (38), the amplitude threshold was increased or reduced by 5% to improve accuracy for certain horse/muscle combinations. Onset, offset, and resultant activity duration for each muscle were normalized to percentage stride duration.

To complement the discrete variables, continuous sEMG data, in the form of amplitude-normalized sEMG signals across all strides/conditions were prepared for analysis. For each horse, high-pass filtered, and full-wave rectified sEMG signals were enveloped using a Butterworth 4th order, low-pass filter (25 Hz cut-off) and were normalized to an RVC. This was the peak amplitude value of the enveloped signals observed for each muscle location across all strides (excluding detected ARV outlier strides) from the corresponding baseline condition. As the RVC represents a submaximal contraction, it was possible for both normalized ARV and continuous data from the iFL/iHL conditions to exceed 100% of the RVC. To ensure that signals were normalized to a peak value that accurately reflects muscular effort, outliers in peak amplitude data were detected from baseline conditions prior to normalization of continuous signals, using the method employed for ARV data. Any additional outlier strides, detected using peak amplitude data, were then excluded from the sEMG dataset.

#### Kinematic signal processing and analysis

Kinematic data were tracked in Qualisys Track Manager (Qualisys AB, Sweden) and imported into Visual3D and Matlab (Version 2020b, The MathWorks Inc., USA) software for further analysis. Gait event detection for stride segmentation and the calculation of stride duration, stride speed, and motion asymmetry variables (MinDiff and MaxDiff of Poll, Pelvis and Withers, Hip Hike) were conducted in Matlab, with joint time-angle data calculated in Visual3D. Hip Hike was calculated as the difference between the upward movement of the left and right tubera coxae during swing phase (47). Stride speed was calculated by smoothed differentiation of the horizontal coordinates (x, y) of the reflective marker between the tubera sacrale. Gait events (hindlimb impact events) were detected in accordance with the method described by Roepstorff et al. (48). These events were manually imported into Visual3D for stride segmentation of sEMG and kinematic data. Motion asymmetry variables were used to quantify lameness and calculated for each stride using upper body vertical displacement data from poll, withers and pelvis markers, which were high-pass filtered (Butterworth 4th order) with a cut-off frequency that was adjusted to the stride frequency of each measurement (49). Asymmetry variables were calculated in accordance with the methods described by Rhodin et al. (17) and Starke et al. (47). Lameness induction was considered sufficient when the motion asymmetry difference between baseline and lameness induction at trot on a straight line surpassed previously described reference values of 13 mm for head movement (MinDiff Poll or MaxDiff Poll) and 5 mm for pelvic motion asymmetry (MinDiff Pelvis and/or MaxDiff Pelvis) and with standard deviations less than their respective means (50).

Continuous angle-time data were calculated in Visual3D for the fore- and hindlimb joints affected by the studied muscles, as well as overall fore- and hindlimb pro-retraction angles. For the forelimb, shoulder and elbow joint angles were calculated. For the hindlimb, hip, stifle and tarsal joint angles were calculated. Kinematic data from the fore- and hindlimb markers were interpolated (maximum gap: 10 frames) and filtered using a Butterworth 4th order low-pass filter, with a 10 Hz cut-off frequency, which was determined using Fast Fourier Transform to ensure that 95% of signal content was retained. For each horse, rigid body models of the forelimb and hindlimb were created. Briefly, virtual landmarks were created 2 cm medial to each anatomical landmark, as described by Hobbs et al. (51), and rigid segments were defined using anatomical and virtual marker coordinates from the static trial. Rigid-body segment models were applied to all dynamic trials from the same horse and joint angles were calculated based on the static trial using the cardan sequence x, y, z. Joint angles were calculated in the sagittal plane, where flexion/extension was defined as rotation around the segment coordinate system x-axis and the flexor side defined as caudal for shoulder and stifle joints, and as cranial for elbow, hip and tarsal joints. To calculate fore- and hindlimb pro- retraction angles, limb positions were defined using a line connecting the proximal end of the spina scapulae to the center of rotation of the metacarpophalangeal joint for the forelimb, and connecting the tuber coxae to the center of rotation of the metatarsophalangeal joint for the hindlimb. Pro- retraction angles were calculated in relation to a body reference position, defined using a line connecting the trunk marker at T6 and the tubera sacrale.

#### Statistical analyses

To increase statistical power, motion asymmetry variables from right iFL and iHL were multiplied by −1 to mirror the indices and thus categorize all data as if they were derived from left fore- and hindlimb inductions only. The remaining variables, including sEMG variables and other data from right iFL and iHL, were also mirrored. Therefore, all results are reported as results of the “lame” side (LS) (ipsilateral to the side of induced lameness) and the “non-lame” side (NLS) (contralateral to the side of induced lameness).

Linear mixed models were used to estimate the effect of lameness induction, with iFL and iHL modeled separately. Stride level data for discrete sEMG and kinematic variables were entered into the model from the baseline measurements and the corresponding induced lameness measurement (baseline 1 and iFL, baseline 2 and iHL) from each horse. Models were calculated in RStudio (Version 3.6.3, RStudio, USA) using the package “lme4” (Version 1.1-15). In each model, horse was used as a random effect and the lameness condition as fixed effect. To evaluate the effect of speed on results, additional, separate analyses were conducted, using speed as a random slope to correct for this variable. Model fit was evaluated using q-q plots and boxplots of the residuals. For each model, results are presented as estimated marginal means, standard error (SE) calculated using the package “emmeans” (Version 1.7.1). Significance values were corrected for multiple comparisons using the false discovery rate method (52).

For the statistical analysis of the continuous kinematic and sEMG data (i.e., complete timeseries of the normalized signals from one stride), statistical parametric mapping (SPM) was used (53, 54). The time and amplitude normalized stride values for sEMG data and the angle-time curves for kinematic data were assembled into 1^*^101^*^1 vector fields (median stride, 101 datapoints per stride and 1 dimension per data point) for each signal, condition, and horse. The open source spm1d package (Version M.0.4.1.) was used to conduct the SPM analysis in Matlab (Version 2020b). For both the sEMG and kinematic data, separate analyses were performed to compare signals between baseline measurements and the corresponding induced lameness measurement (baseline 1 and iFL, baseline 2 and iHL). For sEMG and kinematic data, paired samples *T*-tests were performed on forelimb muscles (triceps, latissimus) and joints (shoulder, elbow, FL pro/retraction), and on hindlimb muscles (biceps, gluteus, semitendinosus) and joints (hip, stifle, hock, hindlimb pro/retraction) together, but separately for the lame side and non-lame side. The two-tailed significance level was set at α = 0.05 and *p*-values were adjusted for multiple comparisons using the Bonferroni correction.

## Results

Appendicular muscle activation and limb movement patterns during trot are presented in Supplementary Video S1, containing the moving 3D model and associated kinematic and sEMG signals from a representative horse during the baseline 1 condition. A total of 647 strides were employed for linear mixed model analysis of discrete kinematic and sEMG data (Baseline 1: 163; Baseline 2: 132: iFL: 189; iHL: 163). A maximum of 243 strides were employed for the separate SPM analysis of continuous sEMG and kinematic data from the LS and NLS fore- and hindlimbs during baseline and corresponding lameness conditions. The proceeding sections include descriptive data (estimated marginal means and SE) and results from the model with a statistical correction for speed (Table 1, Supplementary Table S1). For comparative purposes, results from the non-speed corrected model are presented in Supplementary Tables S2, S3. Throughout this section, all comparisons are between the corresponding baseline and induced lameness conditions.

**Table 1:** Estimated marginal means (EM Mean) and standard error (S.E.) for baseline and lameness induction conditions, and estimated differences (EM Mean Difference, EM Mean % Difference) between corresponding baseline and induction conditions and associated *p*-values for discrete stride duration (s), asymmetry variables (mm), and sEMG ARV variables (%).

**Variable**	**Induction**	**Baseline**		**Induction**		**EM mean difference**	**EM mean % difference**	***p*-value**
		**EM mean**	**S.E**.	**EM mean**	**S.E**.			
Stride duration (s)	iFL	0.77	0.01	0.75	0.01	−0.01	1.30	<0.0001
	iHL	0.73	0.01	0.71	0.01	−0.02	2.74	<0.0001
**Asymmetry variables (mm)**
MinDiff poll	iFL	−3.36	5.30	−57.09	5.22	−53.73	n/a	<0.0001
	iHL	−5.72	5.17	−13.85	5.10	−8.13	n/a	<0.0001
MaxDiff poll	iFL	−7.18	5.38	−29.47	5.72	−22.29	n/a	<0.0001
	iHL	−2.87	3.04	−11.95	2.92	−9.08	n/a	<0.0001
MinDiff withers	iFL	−2.36	1.64	−15.51	1.75	−13.14	n/a	<0.0001
	iHL	−2.07	1.70	10.96	1.72	13.04	n/a	<0.0001
MinDiff pelvis	iFL	1.03	1.14	3.25	1.29	2.22	n/a	<0.0001
	iHL	0.34	2.68	−21.91	2.67	−22.25	n/a	<0.0001
MaxDiff pelvis	iFL	0.68	3.27	6.29	3.28	5.61	n/a	<0.0001
	iHL	4.78	1.37	−23.08	1.39	−27.87	n/a	<0.0001
Hip Hike swing	iFL	0.81	4.22	13.98	4.23	13.17	n/a	<0.0001
	iHL	2.89	6.31	−58.84	6.33	−61.73	n/a	<0.0001
**sEMG ARV (%)**
NLS biceps femoris	iFL	80.94	6.52	112.27	7.38	31.33	38.71	<0.0001
	iHL	43.67	13.16	157.24	11.91	113.57	260.06	<0.0001
LS biceps femoris	iFL	89.53	5.79	96.35	6.04	6.82	7.62	<0.0001
	iHL	75.73	8.52	109.08	9.09	33.36	44.05	<0.0001
NLS superficial gluteal	iFL	67.17	12.95	112.34	13.39	45.17	67.25	<0.0001
	iHL	93.28	6.29	106.85	6.38	13.57	14.55	<0.0001
LS superficial gluteal	iFL	91.00	9.34	116.31	10.56	25.31	27.81	<0.0001
	iHL	98.57	8.82	139.38	9.09	40.82	41.41	<0.0001
NLS semitendinosus	iFL	116.29	44.04	163.90	45.44	47.61	40.94	<0.0001
	iHL	58.53	13.24	113.99	13.18	55.46	94.75	<0.0001
LS semitendinosus	iFL	78.60	8.95	95.75	9.65	17.15	21.82	<0.0001
	iHL	100.83	32.62	189.42	34.20	88.58	87.85	<0.0001
NLS triceps brachii	iFL	76.29	1.67	68.47	1.70	−7.82	10.25	<0.0001
	iHL	78.57	13.96	112.52	14.58	33.95	43.21	<0.0001
LS triceps brachii	iFL	90.44	4.38	112.31	4.82	21.87	24.18	<0.0001
	iHL	89.29	8.94	94.98	8.99	5.70	6.38	0.02
NLS latissimus dorsi	iFL	86.64	4.53	79.42	4.71	−7.23	8.34	<0.0001
	iHL	85.69	4.47	90.75	4.38	5.06	5.91	0.08
LS latissimus dorsi	iFL	92.50	4.42	102.31	4.74	9.81	10.61	<0.0001
	iHL	84.70	4.20	104.40	4.33	19.69	23.25	<0.0001

Data for induced forelimb (iFL) and induced hindlimb (iHL) lameness conditions are presented with each model containing a speed*condition fixed effect. Bilateral sEMG ARV data are presented for each muscle from the non-lame side (NLS) and lame side (LS), based on the side of induced lameness.

### Effect of forelimb lameness induction

**Figure 2:**
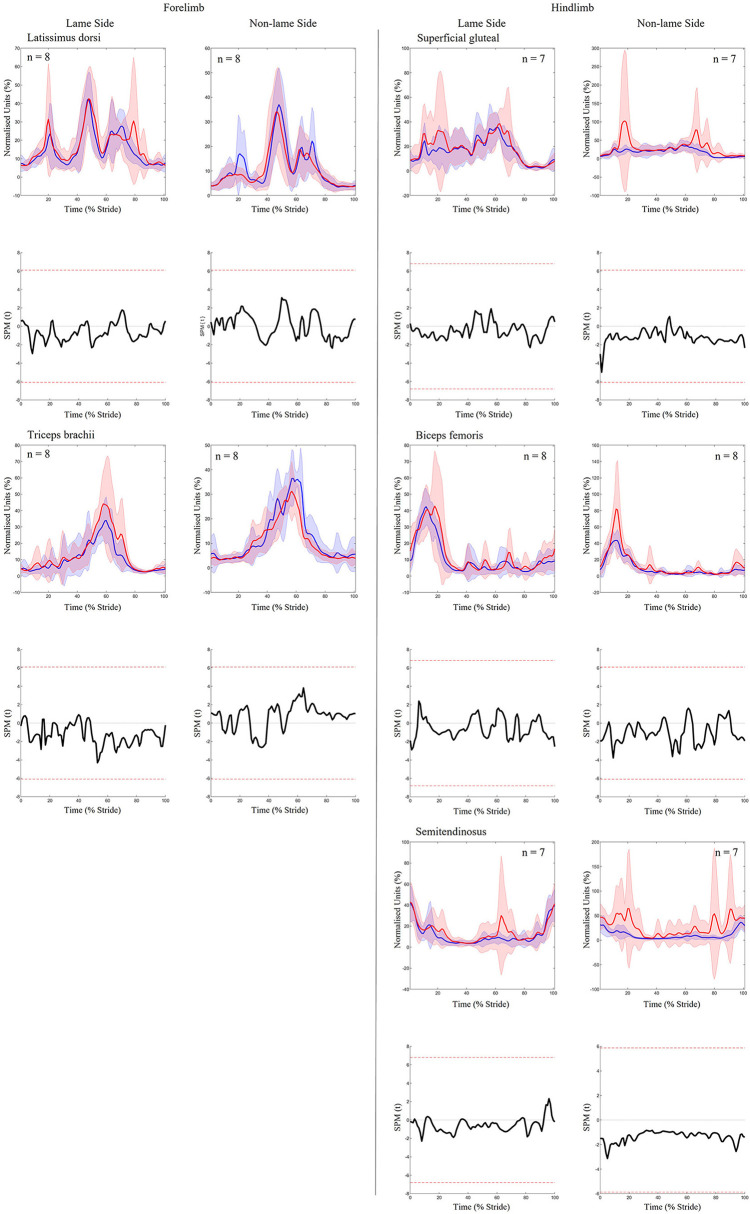
SPM results for time- and amplitude-normalized sEMG data across the group of horses (n = 8) for studied superficial muscles of the forelimb **(Left panel)** and hindlimb **(Right panel)** during baseline 1 (blue solid lines/shaded area) and induced forelimb lameness (red solid lines/shaded area) conditions. sEMG data are DC-offset removed, high-pass filtered (Butterworth 4th order, 40 Hz cut-off), full-wave rectified and enveloped using a low-pass filter (Butterworth, 4th order, 25 Hz cut-off). Within each panel, sEMG data from the lame side and non-lame side are presented on the left and right side graphs, respectively. For each muscle, upper graphs illustrate median (solid line) and standard deviation (shaded area) sEMG data and lower graphs illustrate the paired samples *t*-test SPM result (black solid line) and the critical thresholds for significance (red dashed line). Data are time-normalized between impacts of the contralateral hindlimb. n values for each variable are presented.

**Figure 3:**
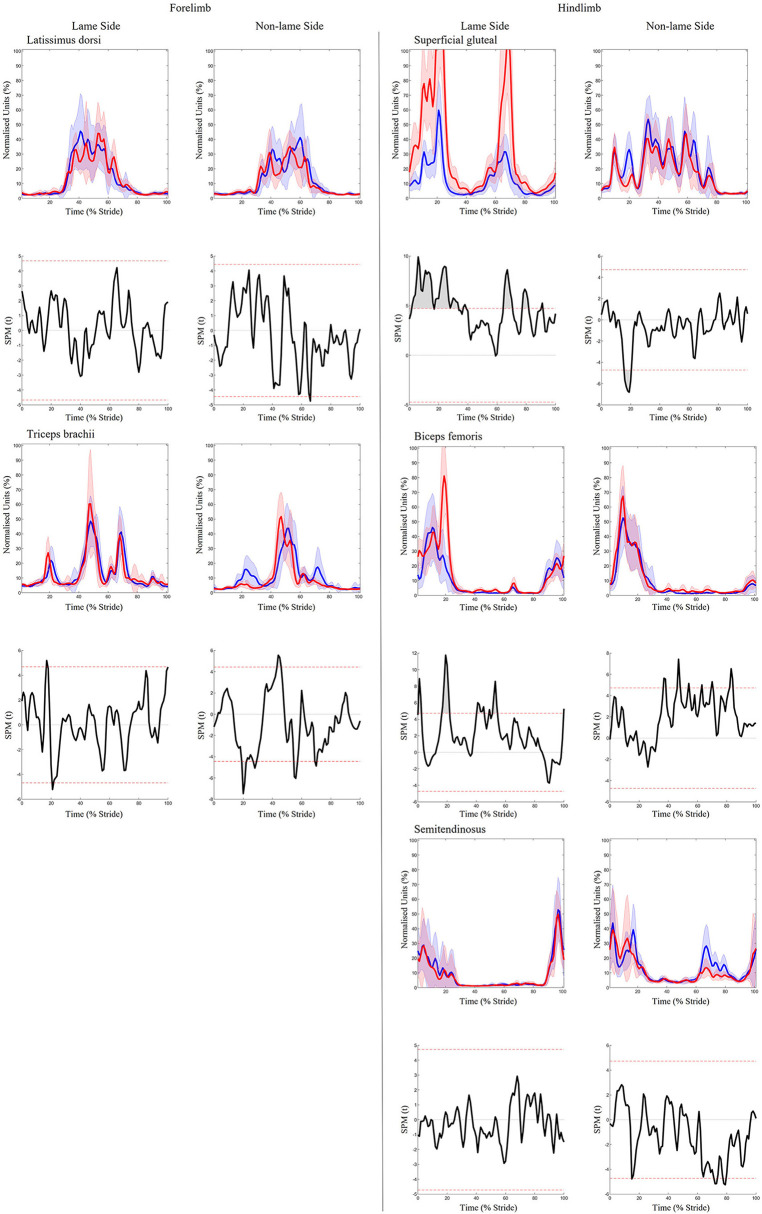
SPM results for time- and amplitude-normalized sEMG data from a representative “horse 1” (*n* = 1) for studied superficial muscles of the forelimb **(Left panel)** and hindlimb **(Right panel)** during baseline 1 (blue solid lines/shaded area) and induced forelimb lameness (red solid lines/shaded area) conditions. sEMG data are DC-offset removed, high-pass filtered (Butterworth 4th order, 40 Hz cut-off), full-wave rectified and enveloped using a low-pass filter (Butterworth, 4th order, 25 Hz cut-off). Within each panel, sEMG data from the lame side and non-lame side are presented on the left and right side graphs, respectively. For each muscle, upper graphs illustrate median (solid line) and standard deviation (shaded area) sEMG data and lower graphs illustrate the paired samples *t*-test SPM result (black solid line) and the critical thresholds for significance (red dashed line). Gray shaded areas indicate regions with statistically significant differences between conditions. Data are time-normalized between impacts of the contralateral hindlimb.

For forelimb muscles (triceps, latissimus), significant decreases in sEMG activity, quantified using ARV, were observed on the NLS, with significant increases observed on the LS (Table 1). Activity duration for triceps was significantly longer on the NLS through significantly earlier onset and later offset events (Supplementary Table S1). On the LS, triceps activity duration was significantly shorter through a significantly later onset event (Supplementary Table S1). Significant increases in ARV, were observed bilaterally across all hindlimb muscles (biceps, gluteal, semitendinosus), with greater increases observed on the NLS (Table 1). A phasic shift in the activation pattern of NLS hindlimb muscles (biceps, gluteal, semitendinosus) was observed through significant delays across onset events within the stride cycle, with NLS semitendinosus also exhibiting significantly delayed offset events (Supplementary Table S1). On the LS, onset of gluteal and semitendinosus, and offset of biceps, occurred significantly earlier (Supplementary Table S1). Results from the non-speed corrected model (Supplementary Tables S2, S3) were similar except for LS latissimus and NLS biceps and gluteal activation events that differed from the speed-corrected model (Supplementary Table S1). SPM results were non-significant when sEMG data were grouped across all horses (Figure 2). However, sEMG waveforms from individual horses showed significant differences between conditions when analyzed using SPM, as illustrated by “horse 1” in Figure 3 and agreed with significant differences in discrete sEMG data (Table 1, Supplementary Table S1). Additional SPM results for sEMG data from “horse 1,” including *p*-values and the beginning and end time points for each data cluster that exceeded the critical thresholds are presented in Supplementary Table S4.

**Figure 4:**
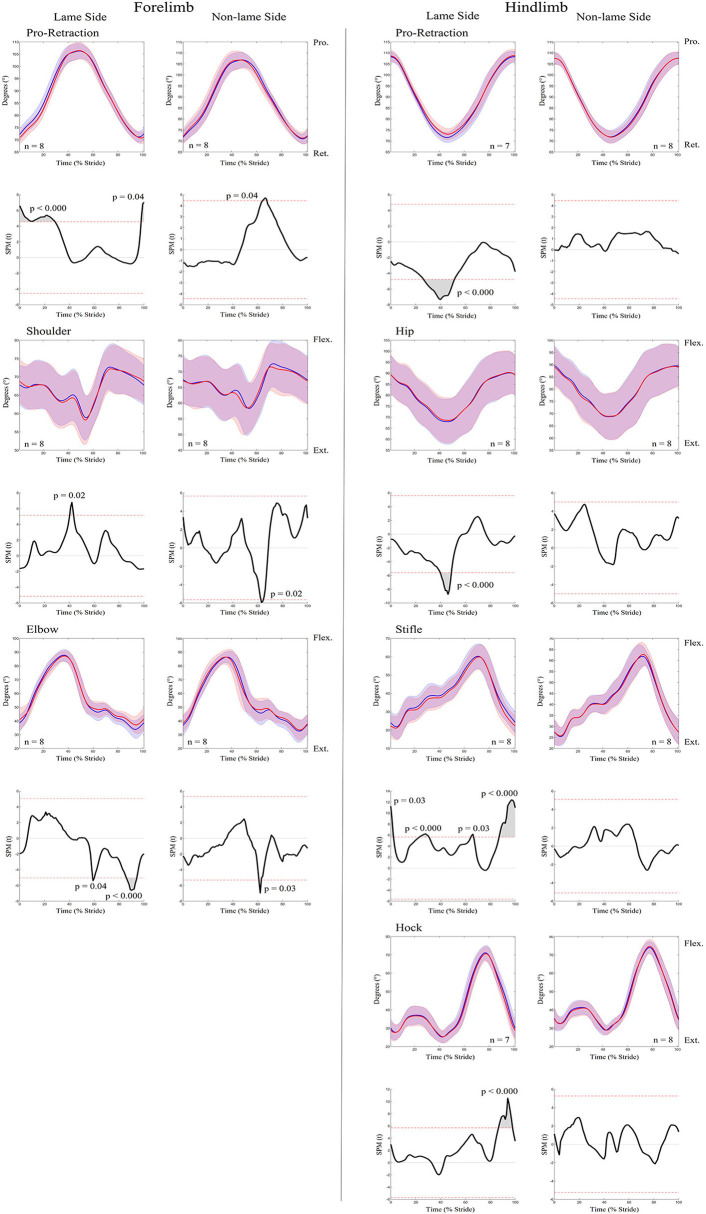
SPM results for time-normalized kinematic data across the group of horses (*n* = 8) for sagittal plane joint angles and pro-retraction angles of the forelimb **(Left panel)** and hindlimb **(Right panel)** during baseline 1 (blue solid lines/shaded area) and induced forelimb lameness (red solid lines/shaded area) conditions. Data are filtered using a Butterworth 4th order low-pass filter, with a 10 Hz cut-off frequency. Within each panel, kinematic data from the lame side and non-lame side are presented on the left and right side graphs, respectively. For each kinematic variable, upper graphs illustrate median (solid line) and standard deviation (shaded area) kinematic data and lower graphs illustrate the paired samples *t*-test SPM result (black solid line) and the critical thresholds for significance (red dashed line). Gray shaded areas indicate regions with statistically significant differences between conditions. *P*-values for each data cluster and n values for each variable are presented. Data are time-normalized between impacts of the contralateral hindlimb.

An increase in most kinematic asymmetry variables was found for iFL (Table 1), including Poll MinDiff (53.73 mm) and Withers MinDiff (13.14 mm). SPM results for kinematic data during iFL are presented in Figure 4 and Table 2 and showed significant differences between conditions for all sagittal plane joint angles and pro-retraction angles of the LS and NLS forelimb, and for the LS hindlimb. LS forelimb protraction was significantly delayed and reduced throughout swing phase and peak retraction was significantly greater and delayed. In contrast, peak protraction of the NLS forelimb occurred significantly earlier in the stride cycle. The LS and NLS shoulder joints extended significantly earlier prior to stance, with a significantly greater peak extension angle occurring on the LS. The LS and NLS elbow joints were less extended throughout stance. In the hindlimbs, significant differences were only observed in the LS, where peak retraction and hip joint extension were significantly decreased during stance. Increased LS stifle and tarsus joint extension occurred significantly earlier during swing phase, with the LS stifle more extended throughout stance.

**Table 2:** Results from SPM analysis of kinematic data, presenting information on the data clusters that exceeded the critical thresholds for significance between baseline and induced lameness (iFL, iHL) conditions.

**Variable**	**Induction**	**Limb**	***n* Clusters**	**Cluster range** **(% stride)**		***p*-value**
				**Start**	**End**	
Hindlimb pro-retraction (°)	iFL	LS	1	26.3	51.1	<0.0001
Hip angle (°)	iFL	LS	1	39.8	48.7	<0.0001
Stifle angle (°)	iFL	LS	4	0.0	2.2	0.03
				23.0	30.7	<0.0001
				64.4	66.5	0.03
				87.1	100.0	<0.0001
Hock angle (°)	iFL	LS	1	86.6	97.9	<0.0001
Forelimb pro-retraction (°)	iFL	LS	2	0.0	28.4	<0.0001
				97.7	100.0	0.04
	iFL	NLS	1	63.9	67.3	0.04
Shoulder angle (°)	iFL	LS	1	40.2	43.4	0.02
	iFL	NLS	1	62.5	64.4	0.02
Elbow angle (°)	iFL	LS	2	58.8	60.0	0.04
				85.9	94.2	<0.0001
	iFL	NLS	1	60.9	62.9	0.03
Hindlimb pro-retraction (°)	iHL	LS	1	22.5	53.7	<0.0001
	iHL	NLS	3	20.4	27.6	0.01
				54.1	71.8	<0.0001
				81.9	98.5	<0.0001
Hip angle (°)	iHL	NLS	2	11.6	14.1	0.02
				86.8	97.4	<0.0001
Stifle angle (°)	iHL	LS	1	29.0	32.8	0.03
Forelimb pro-retraction (°)	iHL	LS	1	52.5	54.5	0.05

Data are presented for kinematic variables from the lame side (LS), and non-lame side (NLS) limbs, where significant differences/data clusters were detected. The number of data clusters identified per kinematic variable (n clusters) are presented. P-values and the beginning and end time points (as a percentage of stride) are presented for each data cluster.

### Effect of hindlimb lameness induction

**Figure 5:**
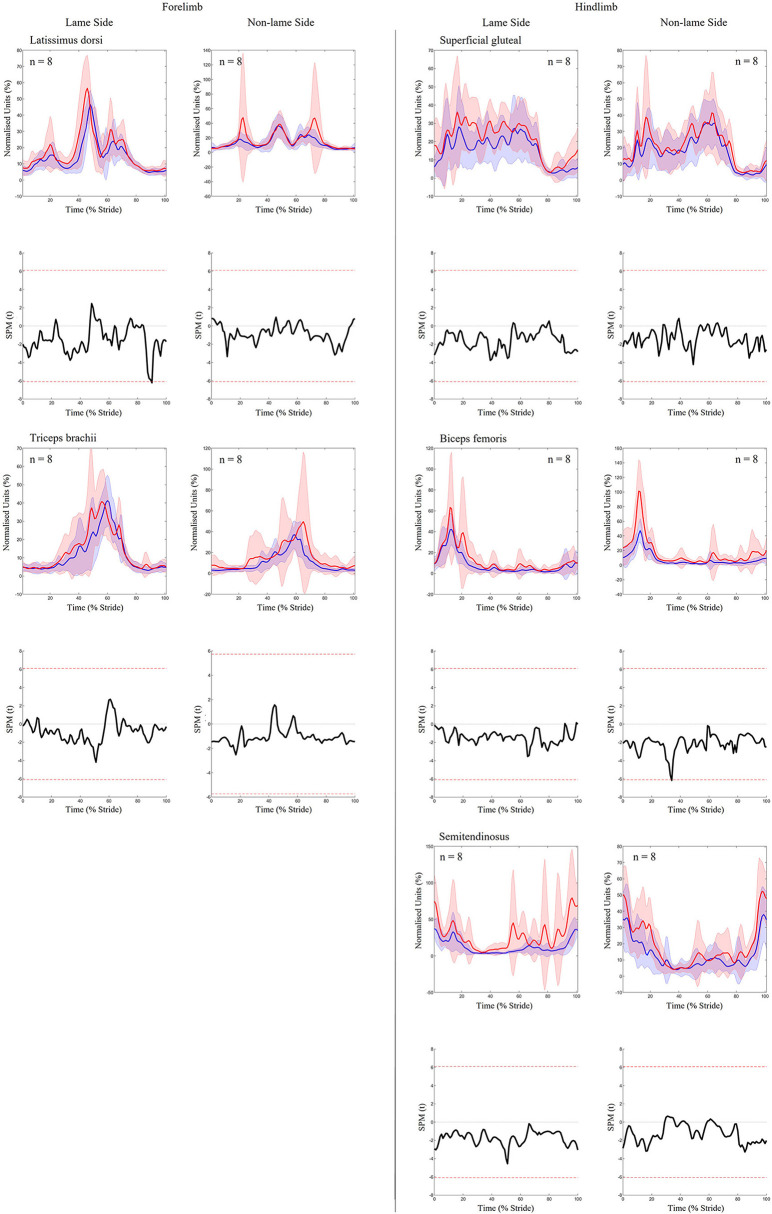
SPM results for time- and amplitude-normalized sEMG data across the group of horses (*n* = 8) for studied superficial muscles of the forelimb **(Left panel)** and hindlimb **(Right panel)** during baseline 1 (blue solid lines/shaded area) and induced hindlimb lameness (red solid lines/shaded area) conditions. sEMG data are DC-offset removed, high-pass filtered (Butterworth 4th order, 40 Hz cut-off), full-wave rectified and enveloped using a low-pass filter (Butterworth, 4th order, 25 Hz cut-off). Within each panel, sEMG data from the lame side and non-lame side are presented on the left and right side graphs, respectively. For each muscle, upper graphs illustrate median (solid line) and standard deviation (shaded area) sEMG data and lower graphs illustrate the paired samples t-test SPM result (black solid line) and the critical thresholds for significance (red dashed line). Data are time-normalized between impacts of the contralateral hindlimb. n values for each variable are presented.

**Figure 6:**
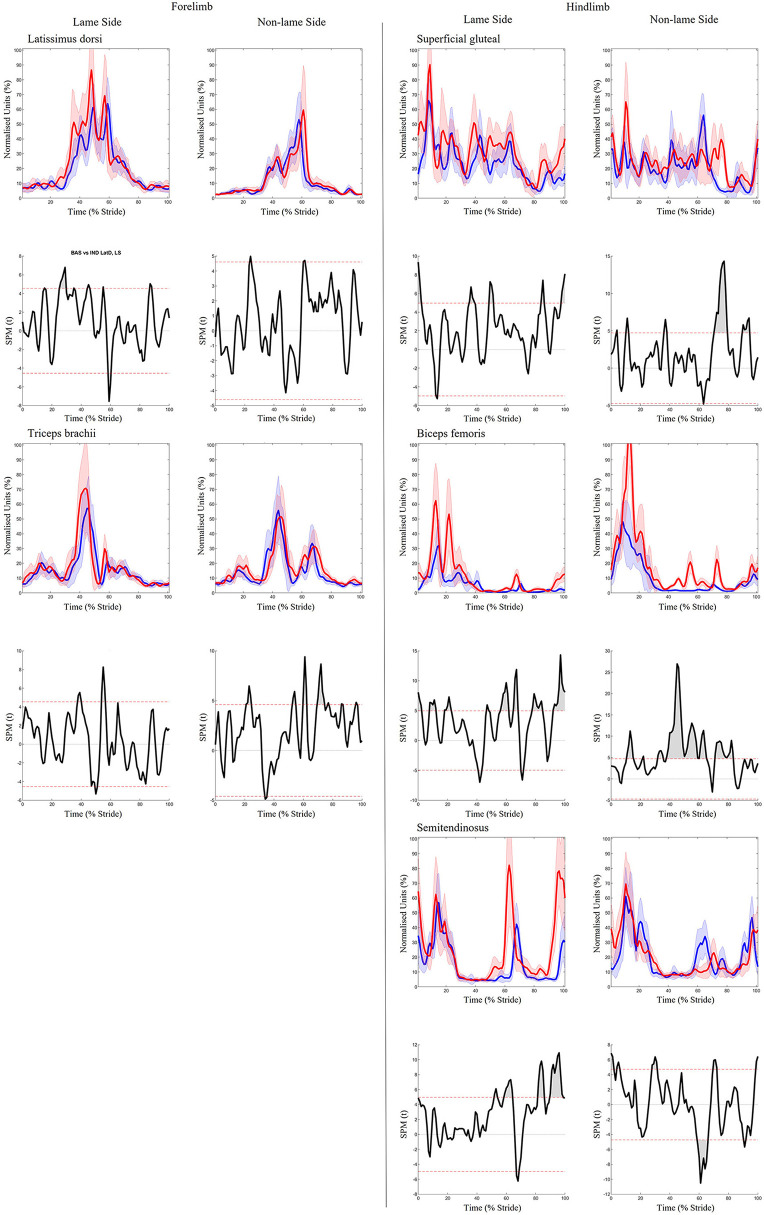
SPM results for time- and amplitude-normalized sEMG data from a representative “horse 7” (*n* = 1) for studied superficial muscles of the forelimb **(Left panel)** and hindlimb **(Right panel)** during baseline 1 (blue solid lines/shaded area) and induced hindlimb lameness (red solid lines/shaded area) conditions. sEMG data are DC-offset removed, high-pass filtered (Butterworth 4th order, 40 Hz cut-off), full-wave rectified and enveloped using a low-pass filter (Butterworth, 4th order, 25 Hz cut-off). Within each panel, sEMG data from the lame side and non-lame side are presented on the left and right side graphs, respectively. For each muscle, upper graphs illustrate median (solid line) and standard deviation (shaded area) sEMG data and lower graphs illustrate the paired samples *t*-test SPM result (black solid line) and the critical thresholds for significance (red dashed line). Gray shaded areas indicate regions with statistically significant differences between conditions. Data are time-normalized between impacts of the contralateral hindlimb.

Significant increases in sEMG ARV were observed bilaterally across all hindlimb muscles (biceps, gluteal, semitendinosus) (Table 1). Muscle activity duration significantly increased for all NLS hindlimb muscles, with gluteal and semitendinosus exhibiting significantly delayed offset events (Supplementary Table S1). On the LS, gluteal and semitendinosus offset, and semitendinosus onset, events occurred significantly earlier (Supplementary Table S1). In the forelimbs, significant increases in ARV were observed bilaterally for triceps, but only on the LS for latissimus (Table 1). Muscle activity duration significantly increased for all NLS forelimb muscles (triceps, latissimus) through significantly delayed offset events (Supplementary Table S1). On the LS, triceps and latissimus offset events occurred significantly earlier, with triceps exhibiting significantly decreased activity duration (Supplementary Table S1). Results from the non-speed corrected model (Supplementary Tables S2, S3) were similar except for NLS latissimus ARV, which was significantly greater during iHL and some LS biceps and NLS gluteal activation events, which differed from the speed corrected model (Table 1, Supplementary Table S1). SPM results were non-significant when sEMG data were grouped across all horses (Figure 5). However, SPM analysis of sEMG waveforms from individual horses revealed significant differences between conditions that agreed with discrete sEMG data (Table 1, Supplementary Table S1), as illustrated by “horse 7” in Figure 6. Additional SPM results for sEMG data from “horse 7,” including p values and the beginning and end time points for each data cluster are presented in Supplementary Table S5.

**Figure 7:**
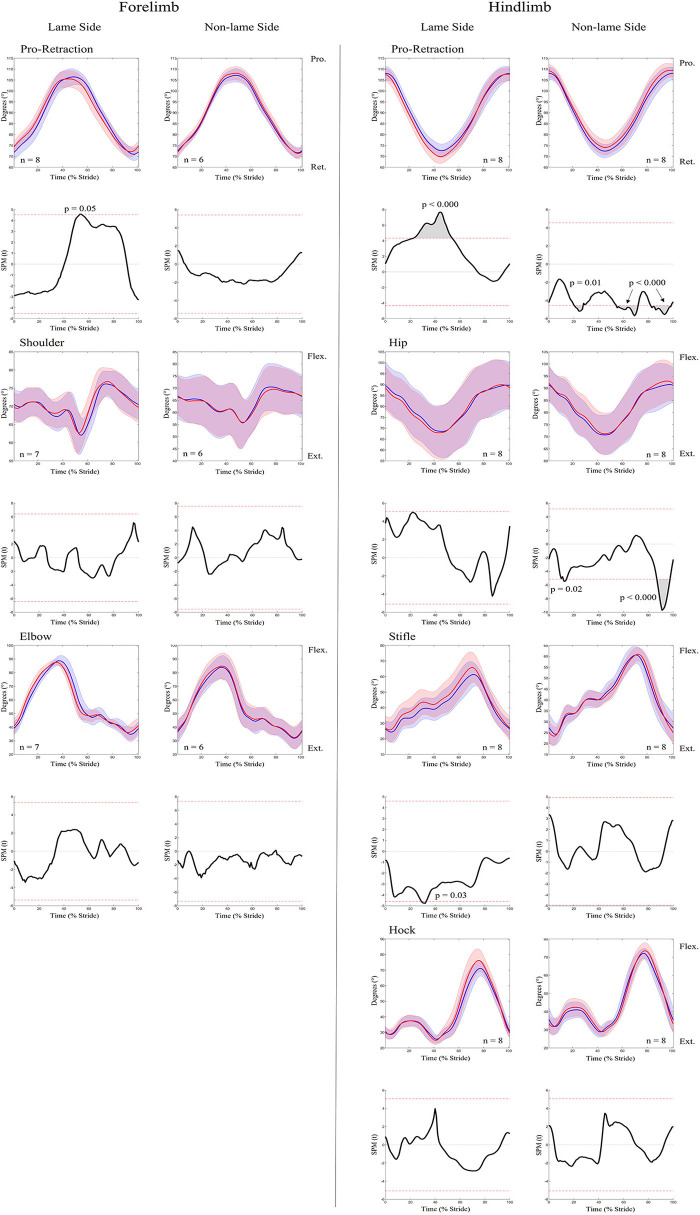
SPM results for time-normalized kinematic data across the group of horses (*n* = 8) for sagittal plane joint angles and pro-retraction angles of the forelimb **(Left panel)** and hindlimb **(Right panel)** during baseline 2 (blue solid lines/shaded area) and induced hindlimb lameness (red solid lines/shaded area) conditions. Data are filtered using a Butterworth 4th order low-pass filter, with a 10 Hz cut-off frequency. Within each panel, kinematic data from the lame side and non-lame side are presented on the left and right side graphs, respectively. For each kinematic variable, upper graphs illustrate median (solid line) and standard deviation (shaded area) kinematic data and lower graphs illustrate the paired samples *t*-test SPM result (black solid line) and the critical thresholds for significance (red dashed line). Gray shaded areas indicate data clusters with statistically significant differences between conditions. *P*-values for each data cluster and n values for each variable are presented. Data are time-normalized between impacts of the contralateral hindlimb.

An increase in most asymmetry variables was found for iHL (Table 1), including pelvis MinDiff (22.25 mm), MaxDiff (27.87 mm), and Hip Hike swing (61.73 mm). SPM results for kinematic data during iHL are presented in Figure 7 and Table 2. In the hindlimbs, LS retraction occurred earlier and was significantly greater following iHL. In contrast, NLS hindlimb retraction significantly decreased and was delayed during stance, but protraction significantly increased and occurred earlier throughout swing phase. The NLS hip joint was significantly more flexed during late swing. Except for short, but significant periods of increased LS stifle flexion and decreased NLS hip extension during stance), no other significant differences between conditions were observed for hindlimb joints during iHL. In the forelimbs, significant differences were only observed for LS peak protraction angle, which decreased and occurred earlier in the stride cycle.

## Discussion

This study combined, for the first time, motion capture and sEMG technology to quantify and compare activity of superficial fore- and hindlimb muscles and associated joint and limb pro-retraction angles during baseline and induced fore- and hindlimb lameness conditions. Findings from this study support the hypotheses that adaptations in muscle activity occur during trotting under iFL and iHL conditions, are identifiable using sEMG, and result in altered temporal and angular kinematics that can characterize the phenomenon in an individual horse when compared to their non-lame condition. Briefly, increases in muscle activation were observed bilaterally across all muscles, except for NLS forelimb muscles during iFL in which activation significantly decreased. Phasic shifts in muscle activation patterns were observed across fore- and hindlimb muscles, which were generally consistent within each limb and reflected phasic shifts/measured changes in the associated limb movement cycle.

### Adaptations in muscle activation and movement of the forelimb during iFL and iHL

We investigated the triceps and latissimus, based on their superficial location (for sEMG), their potential to contribute to joint angle changes, and the fact that they represent two of the largest muscles of the proximal forelimb (55, 56). The measured activation timings for latissimus and triceps are in agreement with previous sEMG studies, which have described the muscles as being active from mid-late swing phase to mid-late stance phase at trot (37, 57–60), with an additional burst often observed for latissimus at the beginning of the swing phase. These timings coincide with the proposed functionality of triceps and latissimus to flex the shoulder during the swing phase, overcome forward inertia and retract the limb prior to hoof impact, as well as their respective anti-gravity roles for stabilizing the elbow and shoulder and controlling movement of the trunk during stance phase (37, 55–60). Thus, the quality of sEMG data within this study, as an accurate representation of latissimus and triceps activity, can be confirmed by agreement with existing equine sEMG literature and descriptions of the functional roles of both muscles.

During iFL, changes in temporal stride patterns are particularly evident in the LS forelimb, where the shortened suspension phase of the lame diagonal pair (1–3, 61) significantly delayed peak pro- and retraction (Figure 4). The result of these adaptations has been described as a “less bouncy” trotting gait during lameness (62–64). To achieve this, a more upright forelimb with more elevated proximal segments, correlates with a more elevated trunk position during LS forelimb stance (65, 66), which is supported by the slight, but largely non-significant, decreases in peak shoulder flexion and elbow extension observed here during lame diagonal stance for both iFL and iHL (Figure 4). Indeed, the proximal structures of the forelimb function as a stiff spring, showing a propensity for generating negative work during stance, where energy absorption has been observed for triceps and postulated for the extrinsic musculature, like latissimus (67, 68). Further, it has been suggested that the extensor musculature of the proximal forelimb joints play an active role in lameness management through active damping efforts to reduce the rate of proximal joint flexion and reduce peak loading during stance phase (4). In this study, we provide the first evidence to support this theory through increased ARV of triceps and latissimus within the lame diagonal pair following iFL and iHL, which may reflect requirements for increased workload to damp vertical forces during lame diagonal stance (4) and to subsequently resist vertical displacement and acceleration of the trunk (3).

The NLS triceps and latissimus were the only muscles to exhibit significant decreases in ARV during iFL. These decreases were seemingly achieved through differing control over the amplitude and duration of muscular response to iFL, with the NLS forelimb muscles exhibiting longer activation bursts of lower amplitude and vice versa for the contralateral LS forelimb. Further, activation of NLS triceps and latissimus occurred significantly earlier during iFL at around 35% of the stride cycle, which corresponded to peak protraction, elbow flexion, and shoulder extension angles. As retractors of the forelimb (37, 55, 56), earlier activation of these muscles represents a functional adaptation to facilitate limb deceleration for earlier impact and subsequent support of the less elevated trunk during a shortened suspension phase (2, 65, 69). Taken together, these findings for NLS forelimb muscles suggests a similar muscular compensatory strategy to that described for mitigating increased vertical impulse of this limb during iFL: by prolonging stance duration and thus the time over which the limb is loaded (2, 64, 69) and muscular force is developed. It is therefore possible that prolonged stance and muscular activation permit the development of adequate muscular force without increased motor unit recruitment or that differing co-activation strategies are employed to mitigate increased activity of triceps and latissimus. However, further work is required to confirm this.

For iHL, visual analysis of Figure 5 reveals increases in the amplitude of LS triceps and latissimus during late swing/early stance phase, and earlier activation onset and offset within the stride cycle. This phasic shift was reflected in kinematic data, facilitating significantly earlier protraction and respective transitions to extension and flexion of the elbow and shoulder joint at around 35% of the stride cycle. Thus, given the clear phasic shifts in muscle activation timings, cyclic limb movements, as well as previous reports of non-significant adaptations in the vertical impulse of the non-lame diagonal forelimb during iHL (1, 62), it seems probable that significant increases in LS triceps and latissimus ARV are related to the facilitation of limb movement adaptations during late swing/early stance phase where the greatest increases in amplitude were observed. Again, further studies which employ synchronized force plate analysis are required to confirm this.

Taken together, our findings from the forelimbs show that, within the lame diagonal pair, significant increases in triceps and latissimus ARV during iFL and iHL are required to stabilize the forelimb against sagittal plane forces, albeit to a seemingly lesser degree during iFL, which we postulate as being related to the greater compensatory role of the head and neck (i.e., “head nod”) for unloading the affected limb (2, 3, 63, 69). Within the non-lame diagonal pair, clear differences in muscular adaptations occurred between iFL and iHL. During iFL, decreases in NLS triceps and latissimus ARV may reflect prolonged increases in the duration of stance and muscular activation, as compensatory strategy for mitigating increased vertical impulse (2, 64, 69). In contrast, a clear phasic shift for earlier activation of LS triceps and latissimus was observed during iHL, which we postulated as being more related to the facilitation of compensatory limb movement than to weightbearing, which has been described as being relatively unaffected in the forelimb (1, 62).

### Adaptations in muscle activation and movement of the hindlimb during iFL and iHL

The hip extensor musculature, including biceps and semitendinosus as studied here, are specialized for force and power generation during stance (70), but also act to stabilize the stifle joint undergoing flexion (67) and to extend the hock (70). In this study, sEMG signals for biceps and semitendinosus conform to previous equine studies that have described their activity from late swing to approximately middle-late stance (34), with semitendinosus exhibiting a second burst from hindlimb lift-off to mid-swing phase (34). Although equine sEMG studies have not evaluated the superficial gluteal, the activation pattern observed here resembled that of semitendinosus and conforms with its proposed functionality as a hip flexor.

During both iFL and iHL, ARV significantly increased across all LS and NLS hindlimb muscles, where increases in amplitude were particularly evident during stance phase (Figure 7). These findings differ from the findings of Zaneb et al. (8), who reported non-significant differences in biceps sEMG activity between a group of non-lame horses and horses with chronic, unilateral hindlimb lameness. Zaneb et al. (8) also reported non-significant differences in peak amplitude for gluteal and semitendinosus muscles across both groups, but found significantly lower amplitude minima:mean ratios (minima vs. mean sEMG amplitude values) in lame horses, which they interpreted as a more distinct “resting phase” of the muscle between contractions. Here, we measured changes in sEMG amplitude across the stride cycle and did not distinguish between amplitude minima and maxima, but significant increases in ARV and general increases in activity duration across LS and NLS hindlimb muscles during iHL (Figure 7) suggest the hindlimb muscles studied here do not exhibit a more distinct “resting phase” between contractions. Different findings can be explained by methodological differences between our studies and Zaneb et al. (8), particularly in relation to the type of lameness (acute/induced vs. chronic cases) and locomotion (overground vs. treadmill), as well as differing sEMG acquisition, processing, and analysis methods.

Buchner et al. (4) reported significant increases in LS and NLS stance phase tarsal joint flexion during iHL, which they attributed to increased damping efforts by the extensor musculature to reduce the rate of vertical limb loading on both hindlimbs. Here, significantly increased ARV of the hip extensors/stifle flexors (biceps, semitendinosus) and hip flexor (gluteal) during iHL, was observed and may therefore reflect increased requirements to stabilize the more loaded NLS limb and mitigate loading of the LS limb over prolonged stance phases (1, 61, 69). Further, significant increases in activity duration occurred across all NLS hindlimb muscles during iHL, through precipitated onset and delayed offset events. Precipitated NLS muscle activation, particularly for the semitendinosus, coincided with significantly earlier and increased NLS hindlimb protraction, as well as subsequent significant increases in NLS hip flexion during late swing (Figure 7). This finding agrees with previous studies that reported a more protracted orientation of the NLS hindlimb, as a means for earlier support of the less elevated trunk (1, 69). Here we provide the first evidence of muscular adaptations underlying these compensatory limb movements. Delayed activation offset of NLS hindlimb muscles may also reflect the generation of greater propulsion during late stance, which has been observed in this limb (71). Thus, increases in ARV observed across hindlimbs during iHL, agree with known adaptations in loading and limb movement that reflect their differing roles as the affected (LS) and compensating (NLS) hindlimb.

To our knowledge, equine hindlimb muscles have not previously been studied during forelimb lameness conditions. Like iHL, we noted significant increases in ARV across all NLS and LS hindlimb muscles during iFL, but with differing phasic activation shifts for delayed NLS activation and precipitated LS activation. Significantly delayed activation across all NLS hindlimb muscles is likely related to the prolonged transition from sound to lame diagonal stance (1, 61, 69), and vice versa for the LS hindlimb muscles which activated earlier. We observed non-significant adaptations in limb movement alongside significant increases in ARV across all NLS hindlimb muscles, which suggests that increased muscular effort is required to stabilize the limb undergoing greater vertical limb loading (2, 64, 69), but also for the generation of greater propulsive forces (64) relative to the contralateral hindlimb. Significantly earlier activation of the LS gluteal and semitendinosus during late swing coincided with significantly earlier and greater extension of the stifle and hock joints (Figures 2, 4). Further, significant decreases in LS hip joint extension during iFL agree with significantly greater retraction, as observed here and by Weishaupt et al. (2). Thus, in a similar vein to the LS forelimb muscles during iHL, increased activation of the LS hindlimb muscles during iFL may be more related to the facilitation of compensatory limb and axial movement than to weightbearing, which has been described as being relatively unaffected in this hindlimb (2, 64, 69).

### Study limitations and further considerations

Given the preliminary nature of this study, there are limitations that must be considered when interpreting our findings. Firstly, an acute lameness induction model was employed to study standardized conditions in a relatively small sample of horses. This transient model served our purpose of exploring the muscular adaptations to lameness and as such is not a limitation. However, the acuteness of the model can be seen as a limitation in the light of the high clinical prevalence of chronic lameness in horses, about which no direct inferences can be made based on the outcome of this study. However, currently no ethically acceptable models of chronic lameness in horses exist and future research employing a larger sample of clinical lameness cases is required as a follow-up to this experimental study. Future studies of this nature should also determine whether the significant changes observed here constitute clinically meaningful changes. In addition, limitations associated with the acquisition and analysis of sEMG data from equine subjects should be considered. sEMG is limited to the evaluation of superficial musculature and only select fore- and hindlimb muscles were studied here, so future studies should evaluate additional muscles. In equine subjects, obtaining a maximum voluntary contraction for normalization purposes is not possible, but normalization to an RVC, as employed here, allowed us to explore the meaningful proportional change in muscle activity between non-lame and induced lameness conditions. Further, normalization of equine sEMG data using an RVC has been shown to improve the sensitivity and accuracy of equine gait analysis (46). ARV was employed as a discrete variable for quantifying changes in sEMG amplitude over the stride cycle (72), but this variable must be cautiously interpreted. This is because changes in sEMG amplitude are related to the recruitment or firing frequency of detected motor units for force generation (20, 43), but caution must be applied when drawing inferences on the relationship between sEMG amplitude and muscular force/contraction type, particularly during dynamic tasks where several intrinsic and extrinsic factors distort this relationship (20, 43). Still, access to time-synchronized kinematic data allowed us to make careful inferences about the relationships between adaptations in movement and muscle activation that occur during iFL and iHL.

The known effect of speed on kinematic and sEMG variables can be considered a limitation of this study, where overground trot speed was not standardized. We addressed this by presenting results from models with- and without a statistical correction for speed (57, 73). Only the adaptations in speed-corrected discrete variables (Table 1, Supplemenatry Table S1) can be considered clinically relevant, as they are not confounded by the effects of speed and are thus the result of induced lameness. To evaluate the effects of induced lameness on appendicular movement, only continuous kinematic data were employed, but a statistical correction for speed could not be applied to the SPM analyses of these data. This could be considered a limitation, however, our SPM results for kinematic data largely agreed with results from other kinematic analyses of induced lameness that standardized speed using a treadmill (1, 2, 4, 74, 75). In contrast to these treadmill studies, that have measured only discrete kinematic variables, our use of continuous kinematic data provides novel information on adaptations in limb movement that occur across the stride cycle during induced, unilateral lameness. Further, the choice for overground locomotion was a deliberate one, as the outcome provides a greater external validity for clinical practice, the lack of which is the main limitation for treadmill studies. This is even more the case when speed is corrected for through appropriate statistical processing.

In this study, SPM results for continuous group-level sEMG data showed non-significant differences, which contrasts results from the linear mixed model analysis of group-averaged discrete data. This could be considered a limitation, but the observed variation in group-level sEMG data and the tighter control of alpha that is required for SPM (53), may explain this discrepancy. This explanation is supported by the fact that, when continuous sEMG data from individual horses were analyzed using SPM, we noted significant differences, which corroborated the results of linear mixed models. We encountered the same discrepancy between discrete and time-series sEMG variables in our study of longissimus activation during induced lameness (76) and this has also been described in previous equine biomechanics research (38, 54, 77). Therefore, in agreement with other researchers (54, 77), we recommend that future studies employ time series data from individual horses when clinically assessing the effects of equine lameness to ensure that subtle changes are not overlooked when considering group-level data.

Up until now, descriptions of compensatory muscular mechanisms that occur during lameness have been largely based on subjective clinical perception. However, findings from this study offer the first objective evidence for underlying muscular adaptations and the associated movements of the fore- and hindlimbs that occur during acute lameness. It is clear from our results that adaptation mechanisms to unilateral lameness occur across all limbs and are primarily related to increased bilateral muscular activity and phasic shifts in activation patterns, which facilitate and/or adapt to pain avoidance mechanisms to reduce loading on the affected limb. The long-term consequences of these muscular adaptations are not known, but further research should investigate this, as prolonged abnormalities in muscular function can manifest as muscle injury or pain (8) and may develop into a chronic condition. Still, the evidence provided here for functional changes in muscle activity during lameness matches with earlier observations and drives home the importance for future sEMG research to further our understanding of these phenomena. The successful combination of sEMG and kinematics in this study may be a small step in the wider field of movement sciences, but it is a great leap forward in equine locomotor research as it opens the possibility to chart and explore, in health and disease, the motion chains in the equine body that underly literally all equestrian disciplines.

## Conclusion

By combining sEMG and motion capture, we identified distinctive differences in appendicular muscle activity that occur during iFL and iHL and this resulted in altered temporal and angular kinematics of the fore- and hindlimbs. The muscular adaptations identified here were primarily related to increased muscular activity, as quantified using ARV, and phasic shifts in activation patterns, which reflected distinctive changes in associated joint angles and phasic shifts of the limb movement cycle. Further studies are required to investigate the clinical meaningfulness of these mechanisms with other lameness causes, in clinical cases, and to better understand the long-term consequences of these muscular adaptations. Still, the successful combination of sEMG and kinematics can be used to further our understanding of the clinical signs and etiology of equine lameness, and with further development, sEMG has the potential to aid in the diagnosis, treatment, and management strategies for lameness.

## Data availability statement

The raw data supporting the conclusions of this article will be made available by the authors, without undue reservation.

## Ethics statement

The animal study was reviewed and approved by Utrecht University (CCD: AVD108002015307) and the University of Central Lancashire (Reference Number: RE/17/08a_b).

## Author contributions

LS, TS, SJH, and FS contributed to conception and design of the study. LS, TS, and FS conducted data collection. LS, SJH, and FS conducted data processing and analysis. FS and IS performed the statistical analysis. LS and TS wrote the first draft of the manuscript. FS and IS wrote sections of the manuscript. All authors contributed to manuscript revision, read, and approved the submitted version.

## Funding

This study was made possible with support from Morris Animal Foundation (Grant ID: D21EQ-406) and the British Society of Animal Science (BSAS) 2018 Steve Bishop Early Career Award. Open Access publication fees were funded by the University of Central Lancashire.

## Conflict of interest

Author SR is employed by Delsys Inc., the manufacturers of the sEMG sensors used in this study. The remaining authors declare that the research was conducted in the absence of any commercial or financial relationships that could be construed as a potential conflict of interest.

## Publisher's note

All claims expressed in this article are solely those of the authors and do not necessarily represent those of their affiliated organizations, or those of the publisher, the editors and the reviewers. Any product that may be evaluated in this article, or claim that may be made by its manufacturer, is not guaranteed or endorsed by the publisher.
