# Supplementary material for: Adaptations in equine appendicular muscle activity and movement occur during induced fore- and hindlimb lameness: An electromyographic and kinematic evaluation
Source: Front Vet Sci. 2022 Nov 8;9:989522. doi: 10.3389/fvets.2022.989522 (PMC9679508; doi:10.3389/fvets.2022.989522)
Supplement: Supplementary file 2 [file Data_Sheet_1.docx]

Supplementary Material

Supplementary Video 1 (SV1): Video showing appendicular movement and muscle activity from one representative horse and stride at trot during the baseline 1 condition. sEMG signals from the right latissimus dorsi, triceps brachii, superficial gluteal, biceps femoris, and right semitendinosus are presented as separate graphs in the right panel, alongside the moving three-dimensional model in the middle panel, to illustrate muscle activation in relation to movement during one trot stride. In a second clip, joint angles from the fore- (shoulder, elbow) and hindlimbs (hip, stifle, hock) joints, which the studied muscles work on, are presented alongside the moving three-dimensional model. Video clips were exported and adapted from Visual3D (C-motion Inc., USA) software.

Table S1. Estimated marginal means (EM Mean) and standard error (S.E.) for baseline and lameness induction conditions, and estimated differences (EM Mean Difference, EM Mean % Difference) between corresponding baseline and induction conditions and associated p-values for discrete sEMG activation onset, offset and activity duration variables (% stride). Data for induced forelimb (iFL) and induced hindlimb (iHL) lameness conditions are presented with each model containing a speed*condition fixed effect. Bilateral sEMG data are presented for each muscle from the non-lame side (NLS) and lame side (LS), based on the side of induced lameness.

| **Variable** | **Induction** | **Baseline** | | **Induction** | | **EM Mean Difference** | **EM Mean % Difference** | **p-value** |
| --- | --- | --- | --- | --- | --- | --- | --- | --- |
|  |  | EM Mean | S.E. | EM Mean | S.E. |  |  |  |
| NLS Biceps Femoris (% Stride) | | | | | | | | |
| Offset | iFL | 32.83 | 1.78 | 30.98 | 1.71 | -1.85 | 5.64 | 0.07 |
|  | iHL | 29.57 | 2.88 | 31.43 | 2.89 | 1.86 | 6.29 | 0.05 |
| Onset | iFL | 91.32 | 1.67 | 93.02 | 1.72 | 1.69 | 1.85 | 0.01 |
|  | iHL | 96.16 | 2.50 | 95.55 | 2.49 | -0.61 | 0.63 | 0.45 |
| Activity Duration | iFL | 49.30 | 2.56 | 41.93 | 2.45 | -7.37 | 14.95 | <0.0001 |
|  | iHL | 32.02 | 4.16 | 41.18 | 4.16 | 9.16 | 28.61 | <0.0001 |
| LS Biceps Femoris (% Stride) | | | | | | | | |
| Offset | iFL | 29.98 | 1.84 | 28.38 | 1.74 | -1.6 | 5.34 | 0.01 |
|  | iHL | 26.12 | 1.87 | 27.84 | 1.96 | 1.72 | 6.58 | 0.07 |
| Onset | iFL | 92.79 | 1.72 | 91.51 | 1.68 | -1.29 | 1.39 | 0.05 |
|  | iHL | 92.44 | 1.39 | 91.22 | 1.39 | -1.22 | 1.32 | 0.20 |
| Activity Duration | iFL | 44.11 | 3.72 | 48.05 | 3.84 | 3.95 | 8.95 | 0.02 |
|  | iHL | 47.43 | 4.75 | 43.57 | 4.74 | -3.86 | 8.14 | 0.06 |
| NLS Superficial Gluteal (% Stride) | | | | | | | | |
| Offset 1 | iFL | 27.47 | 2.57 | 29.19 | 2.60 | 1.72 | 6.26 | 0.37 |
|  | iHL | 31.13 | 2.94 | 32.08 | 3.05 | 0.95 | 3.05 | 0.58 |
| Onset 1 | iFL | 44.93 | 4.58 | 48.67 | 4.52 | 3.74 | 8.32 | 0.04 |
|  | iHL | 40.15 | 3.22 | 42.19 | 3.34 | 2.04 | 5.08 | 0.24 |
| Offset 2 | iFL | 75.03 | 0.95 | 75.89 | 1.04 | 0.86 | 1.15 | 0.20 |
|  | iHL | 76.55 | 0.97 | 78.07 | 0.98 | 1.52 | 1.99 | 0.04 |
| Onset 2 | iFL | 89.27 | 7.13 | 95.15 | 7.20 | 5.89 | 6.60 | 0.02 |
|  | iHL | 91.56 | 7.56 | 90.98 | 7.70 | -0.58 | 0.63 | 0.84 |
| Activity Duration | iFL | 69.62 | 3.61 | 70.24 | 3.56 | 0.61 | 0.88 | 0.68 |
|  | iHL | 69.58 | 5.32 | 72.33 | 5.30 | 2.75 | 3.95 | 0.03 |
| LS Superficial Gluteal (% Stride) | | | | | | | | |
| Offset 1 | iFL | 27.44 | 3.43 | 28.71 | 3.52 | 1.26 | 4.59 | 0.60 |
|  | iHL | 33.99 | 19.85 | 26.11 | 18.93 | -7.88 | 23.18 | 0.97 |
| Onset 1 | iFL | 38.98 | 5.12 | 38.18 | 5.12 | -0.80 | 2.05 | 0.69 |
|  | iHL | 43.61 | 21.30 | 32.53 | 23.33 | -11.08 | 25.41 | 0.97 |
| Offset 2 | iFL | 75.92 | 0.89 | 75.18 | 0.95 | -0.73 | 0.96 | 0.16 |
|  | iHL | 74.91 | 1.23 | 72.62 | 1.23 | -2.30 | 3.07 | <0.0001 |
| Onset 2 | iFL | 99.05 | 5.23 | 90.37 | 5.98 | -8.69 | 8.77 | <0.0001 |
|  | iHL | 97.15 | 3.60 | 93.40 | 3.85 | -3.76 | 3.87 | 0.24 |
| Activity Duration | iFL | 69.02 | 2.89 | 61.86 | 3.18 | -7.17 | 10.39 | <0.0001 |
|  | iHL | 69.90 | 3.59 | 75.38 | 3.58 | 5.47 | 7.83 | <0.0001 |
| NLS Semitendinosus (% Stride) | | | | | | | | |
| Offset 1 | iFL | 18.84 | 1.90 | 22.85 | 1.92 | 4.01 | 21.28 | <0.0001 |
|  | iHL | 19.26 | 2.66 | 23.94 | 2.61 | 4.69 | 24.35 | <0.0001 |
| Onset 1 | iFL | 48.92 | 4.13 | 60.08 | 3.50 | 11.16 | 22.81 | <0.0001 |
|  | iHL | 53.65 | 3.37 | 51.22 | 3.33 | -2.43 | 4.53 | 0.05 |
| Offset 2 | iFL | 65.73 | 2.90 | 70.43 | 2.13 | 4.70 | 7.15 | 0.02 |
|  | iHL | 63.08 | 5.59 | 63.70 | 5.62 | 0.62 | 0.98 | 0.74 |
| Onset 2 | iFL | 85.47 | 2.58 | 86.88 | 2.62 | 1.40 | 1.64 | 0.01 |
|  | iHL | 89.26 | 0.90 | 87.03 | 0.90 | -2.23 | 2.50 | 0.03 |
| Activity Duration | iFL | 39.60 | 4.87 | 44.94 | 4.79 | 5.34 | 13.48 | <0.0001 |
|  | iHL | 33.80 | 4.06 | 44.92 | 4.03 | 11.12 | 32.90 | <0.0001 |
| LS Semitendinosus (% Stride) | | | | | | | | |
| Offset 1 | iFL | 19.90 | 3.25 | 19.11 | 3.20 | -0.79 | 3.97 | 0.44 |
|  | iHL | 24.31 | 1.91 | 20.52 | 1.98 | -3.79 | 15.59 | <0.0001 |
| Onset 1 | iFL | 54.52 | 2.47 | 54.88 | 2.47 | 0.36 | 0.66 | 0.69 |
|  | iHL | 58.30 | 2.37 | 53.05 | 2.39 | -5.25 | 9.01 | <0.0001 |
| Offset 2 | iFL | 71.80 | 3.17 | 76.35 | 3.22 | 4.55 | 6.34 | <0.0001 |
|  | iHL | 74.87 | 1.72 | 69.98 | 1.56 | -4.89 | 6.53 | <0.0001 |
| Onset 2 | iFL | 89.13 | 1.63 | 87.33 | 1.67 | -1.79 | 2.01 | <0.0001 |
|  | iHL | 93.71 | 1.19 | 88.19 | 1.16 | -5.51 | 5.88 | <0.0001 |
| Activity Duration | iFL | 46.27 | 7.37 | 46.36 | 7.38 | 0.10 | 0.22 | 0.96 |
|  | iHL | 42.80 | 5.13 | 43.48 | 5.03 | 0.68 | 1.59 | 0.77 |
| NLS Triceps Brachii (% Stride) | | | | | | | | |
| Onset | iFL | 35.41 | 2.11 | 29.16 | 2.22 | -6.25 | 17.65 | <0.0001 |
|  | iHL | 39.00 | 3.03 | 32.97 | 3.02 | -6.03 | 15.46 | <0.0001 |
| Offset | iFL | 72.45 | 2.23 | 75.19 | 2.21 | 2.74 | 3.78 | <0.0001 |
|  | iHL | 70.68 | 1.19 | 77.64 | 1.20 | 6.97 | 9.86 | <0.0001 |
| Activity Duration | iFL | 40.47 | 2.90 | 47.94 | 3.12 | 7.47 | 18.46 | <0.0001 |
|  | iHL | 30.58 | 3.85 | 47.26 | 3.81 | 16.68 | 54.55 | <0.0001 |
| LS Triceps Brachii (% Stride) | | | | | | | | |
| Onset | iFL | 36.96 | 2.56 | 41.58 | 2.55 | 4.61 | 12.47 | <0.0001 |
|  | iHL | 38.00 | 3.55 | 37.19 | 3.55 | -0.81 | 2.13 | 0.27 |
| Offset | iFL | 72.81 | 1.43 | 72.46 | 1.44 | -0.35 | 0.48 | 0.60 |
|  | iHL | 76.94 | 0.88 | 73.05 | 0.88 | -3.88 | 5.04 | <0.0001 |
| Activity Duration | iFL | 40.33 | 3.07 | 36.80 | 3.16 | -3.53 | 8.75 | 0.01 |
|  | iHL | 44.61 | 3.06 | 41.58 | 3.18 | -3.03 | 6.79 | <0.0001 |
| NLS Latissimus Dorsi (% Stride) | | | | | | | | |
| Onset 1 | iFL | 11.56 | 1.13 | 10.81 | 1.07 | -0.75 | 6.49 | 0.16 |
|  | iHL | 10.72 | 1.15 | 10.31 | 1.20 | -0.41 | 3.82 | 0.51 |
| Offset 1 | iFL | 24.20 | 0.86 | 23.42 | 0.86 | -0.77 | 3.18 | 0.17 |
|  | iHL | 24.31 | 1.58 | 29.60 | 1.96 | 5.29 | 21.76 | <0.0001 |
| Onset 2 | iFL | 38.01 | 1.01 | 36.87 | 1.02 | -1.14 | 3.00 | 0.02 |
|  | iHL | 37.51 | 1.63 | 42.21 | 1.79 | 4.70 | 12.53 | <0.0001 |
| Offset 2 | iFL | 79.98 | 2.01 | 80.34 | 2.09 | 0.36 | 0.45 | 0.60 |
|  | iHL | 76.75 | 0.76 | 82.55 | 0.76 | 5.80 | 7.56 | <0.0001 |
| Activity Duration | iFL | 54.87 | 4.23 | 55.86 | 4.31 | 1.00 | 1.82 | 0.55 |
|  | iHL | 53.61 | 1.79 | 64.56 | 1.76 | 10.94 | 20.41 | <0.0001 |
| LS Latissimus Dorsi (% Stride) | | | | | | | | |
| Onset 1 | iFL | 10.04 | 1.19 | 8.87 | 1.10 | -1.17 | 11.65 | 0.02 |
|  | iHL | 10.94 | 1.58 | 9.33 | 1.54 | -1.61 | 14.72 | 0.01 |
| Offset 1 | iFL | 25.58 | 1.68 | 24.28 | 1.70 | -1.30 | 5.08 | 0.03 |
|  | iHL | 26.52 | 1.69 | 22.04 | 1.70 | -4.48 | 16.89 | <0.0001 |
| Onset 2 | iFL | 37.78 | 1.48 | 36.83 | 1.32 | -0.96 | 2.54 | 0.14 |
|  | iHL | 41.77 | 2.14 | 35.02 | 2.09 | -6.75 | 16.16 | <0.0001 |
| Offset 2 | iFL | 80.37 | 1.65 | 80.20 | 1.74 | -0.16 | 0.20 | 0.78 |
|  | iHL | 80.18 | 1.69 | 74.85 | 1.71 | -5.33 | 6.65 | <0.0001 |
| Activity Duration | iFL | 56.77 | 3.43 | 54.00 | 3.44 | -2.77 | 4.88 | 0.10 |
|  | iHL | 52.20 | 4.04 | 49.91 | 3.99 | -2.29 | 4.39 | 0.23 |

Table S2. Estimated marginal means (EM Mean) and standard error (S.E.) for baseline and lameness induction conditions, and estimated differences (EM Mean Difference, EM Mean % Difference) between corresponding baseline and induction conditions and associated p-values for discrete stride speed (m/s), stride duration (s), asymmetry variables (mm), and sEMG ARV variables (%). Data for induced forelimb (iFL) and induced hindlimb (iHL) lameness conditions are presented. Models do not contain a fixed effect for speed. Bilateral sEMG ARV data are presented for each muscle from the non-lame side (NLS) and lame side (LS), based on the side of induced lameness.

| **Variable** | **Induction** | **Baseline** | | **Induction** | | **EM Mean Difference** | **EM Mean % Difference** | **p-value** |
| --- | --- | --- | --- | --- | --- | --- | --- | --- |
|  |  | EM Mean | S.E. | EM Mean | S.E. |  |  |  |
| Stride Speed (m/s) | iFL | 3.13 | 0.10 | 2.87 | 0.10 | -0.26 | 8.31 | <0.0001 |
|  | iHL | 3.09 | 0.12 | 3.03 | 0.12 | -0.06 | 1.94 | 0.02 |
| Stride Duration (s) | iFL | 0.74 | 0.02 | 0.78 | 0.02 | 0.04 | 5.41 | <0.0001 |
|  | iHL | 0.75 | 0.01 | 0.74 | 0.01 | -0.01 | 1.33 | <0.0001 |
| Asymmetry Variables (mm) | | | | | | | | |
| MinDiff Poll | iFL | -3.99 | 4.42 | -57.35 | 4.41 | -53.36 | n/a | <0.0001 |
|  | iHL | -3.04 | 3.19 | -14.28 | 3.09 | -11.25 | n/a | <0.0001 |
| MaxDiff Poll | iFL | -4.62 | 6.26 | -24.27 | 6.25 | -19.65 | n/a | <0.0001 |
|  | iHL | -3.40 | 2.14 | -13.07 | 2.04 | -9.68 | n/a | <0.0001 |
| MinDiff Withers | iFL | -1.97 | 2.06 | -14.13 | 2.06 | -12.16 | n/a | <0.0001 |
|  | iHL | -2.61 | 1.75 | 11.23 | 1.73 | 13.84 | n/a | <0.0001 |
| MinDiff Pelvis | iFL | -0.65 | 1.75 | 0.79 | 1.75 | 1.44 | n/a | 0.04 |
|  | iHL | 1.40 | 2.13 | -21.24 | 2.11 | -22.64 | n/a | <0.0001 |
| MaxDiff Pelvis | iFL | 3.46 | 1.34 | 9.31 | 1.32 | 5.85 | n/a | <0.0001 |
|  | iHL | 5.60 | 2.55 | -25.74 | 2.52 | -31.34 | n/a | <0.0001 |
| Hip Hike Swing | iFL | 3.92 | 2.33 | 16.72 | 2.32 | 12.80 | n/a | <0.0001 |
|  | iHL | 7.51 | 5.03 | -56.32 | 4.98 | -63.83 | n/a | <0.0001 |
| sEMG ARV (%) | | | | | | | | |
| NLS  Biceps Femoris | iFL | 81.62 | 8.75 | 112.49 | 8.78 | 30.87 | 37.82 | <0.0001 |
|  | iHL | 70.10 | 20.22 | 182.09 | 20.03 | 111.99 | 159.76 | <0.0001 |
| LS  Biceps Femoris | iFL | 79.53 | 4.89 | 84.15 | 4.86 | 4.61 | 5.80 | 0.02 |
|  | iHL | 74.69 | 9.73 | 112.93 | 9.74 | 38.25 | 51.21 | <0.0001 |
| NLS Superficial Gluteal | iFL | 83.68 | 17.54 | 133.23 | 17.42 | 49.55 | 59.21 | <0.0001 |
|  | iHL | 86.23 | 5.17 | 97.34 | 5.14 | 11.11 | 12.88 | <0.0001 |
| LS Superficial Gluteal | iFL | 82.01 | 9.27 | 105.08 | 9.35 | 23.07 | 28.13 | <0.0001 |
|  | iHL | 83.20 | 6.37 | 122.76 | 6.37 | 39.55 | 47.54 | <0.0001 |
| NLS Semitendinosus | iFL | 146.05 | 72.28 | 198.39 | 72.24 | 52.34 | 35.84 | <0.0001 |
|  | iHL | 69.72 | 11.58 | 126.10 | 11.51 | 56.38 | 80.87 | <0.0001 |
| LS Semitendinosus | iFL | 75.93 | 9.13 | 93.55 | 9.22 | 17.62 | 23.21 | <0.0001 |
|  | iHL | 79.21 | 24.98 | 167.22 | 25.01 | 88.01 | 111.11 | <0.0001 |
| NLS  Triceps Brachii | iFL | 78.07 | 1.73 | 67.67 | 1.72 | -10.39 | 13.31 | <0.0001 |
|  | iHL | 72.11 | 19.13 | 122.18 | 19.09 | 50.06 | 69.42 | <0.0001 |
| LS  Triceps Brachii | iFL | 78.51 | 3.88 | 98.50 | 3.89 | 19.98 | 25.45 | <0.0001 |
|  | iHL | 82.63 | 5.12 | 89.61 | 5.12 | 6.97 | 8.44 | <0.0001 |
| NLS Latissimus  Dorsi | iFL | 84.37 | 3.13 | 75.24 | 3.12 | -9.14 | 10.83 | <0.0001 |
|  | iHL | 86.68 | 11.26 | 104.14 | 11.24 | 17.46 | 20.14 | <0.0001 |
| LS Latissimus Dorsi | iFL | 83.46 | 5.10 | 91.74 | 5.10 | 8.28 | 9.92 | <0.0001 |
|  | iHL | 82.17 | 3.28 | 102.24 | 3.28 | 20.07 | 24.42 | <0.0001 |

Table S3. Estimated marginal means (EM Mean) and standard error (S.E.) for baseline and lameness induction conditions, and estimated differences (EM Mean Difference, EM Mean % Difference) between corresponding baseline and induction conditions and associated p-values for discrete sEMG activation onset, offset and activity duration variables (% stride). Data for induced forelimb (iFL) and induced hindlimb (iHL) lameness conditions are presented. Models do not contain a fixed effect for speed. Bilateral sEMG data are presented for each muscle from the non-lame side (NLS) and lame side (LS), based on the side of induced lameness.

| **Variable** | **Induction** | **Baseline** | | **Induction** | | **EM Mean Difference** | **EM Mean % Difference** | **p-value** |
| --- | --- | --- | --- | --- | --- | --- | --- | --- |
|  |  | EM Mean | S.E. | EM Mean | S.E. |  |  |  |
| NLS Biceps Femoris (% Stride) | | | | | | | | |
| Offset | iFL | 31.73 | 1.28 | 29.13 | 1.31 | -2.60 | 8.19 | 0.01 |
|  | iHL | 26.38 | 1.80 | 28.03 | 1.79 | 1.64 | 6.22 | 0.06 |
| Onset | iFL | 92.16 | 1.72 | 94.08 | 1.72 | 1.92 | 2.08 | <0.0001 |
|  | iHL | 95.08 | 1.87 | 95.20 | 1.86 | 0.12 | 0.13 | 0.91 |
| Activity Duration | iFL | 49.77 | 2.93 | 40.93 | 2.95 | -8.84 | 17.76 | <0.0001 |
|  | iHL | 31.60 | 2.77 | 39.88 | 2.75 | 8.28 | 26.20 | <0.0001 |
| LS Biceps Femoris (% Stride) | | | | | | | | |
| Offset | iFL | 30.45 | 1.79 | 28.96 | 1.78 | -1.49 | 4.89 | 0.01 |
|  | iHL | 29.84 | 2.19 | 31.15 | 2.2 | 1.32 | 4.42 | 0.12 |
| Onset | iFL | 92.49 | 1.47 | 91.16 | 1.46 | -1.32 | 1.43 | 0.03 |
|  | iHL | 93.50 | 1.77 | 91.53 | 1.77 | -1.98 | 2.12 | 0.01 |
| Activity Duration | iFL | 44.46 | 3.19 | 48.26 | 3.16 | 3.80 | 8.55 | 0.01 |
|  | iHL | 44.84 | 2.55 | 45.94 | 2.56 | 1.10 | 2.45 | 0.58 |
| NLS Superficial Gluteal (% Stride) | | | | | | | | |
| Offset 1 | iFL | 27.38 | 2.11 | 29.19 | 2.12 | 1.81 | 6.61 | 0.31 |
|  | iHL | 31.03 | 2.38 | 31.11 | 2.46 | 0.08 | 0.26 | 0.97 |
| Onset 1 | iFL | 42.33 | 3.85 | 46.19 | 3.88 | 3.85 | 9.10 | 0.03 |
|  | iHL | 40.37 | 2.58 | 42.18 | 2.67 | 1.81 | 4.48 | 0.13 |
| Offset 2 | iFL | 76.14 | 0.99 | 77.40 | 0.97 | 1.26 | 1.65 | 0.04 |
|  | iHL | 76.04 | 0.79 | 77.24 | 0.77 | 1.20 | 1.58 | 0.08 |
| Onset 2 | iFL | 91.02 | 6.25 | 97.16 | 6.20 | 6.13 | 6.73 | 0.01 |
|  | iHL | 89.26 | 4.87 | 92.62 | 4.78 | 3.36 | 3.76 | 0.29 |
| Activity Duration | iFL | 68.11 | 2.85 | 67.00 | 2.82 | -1.11 | 1.63 | 0.43 |
|  | iHL | 70.47 | 2.70 | 73.45 | 2.68 | 2.98 | 4.23 | 0.01 |
| LS Superficial Gluteal (% Stride) | | | | | | | | |
| Offset 1 | iFL | 27.26 | 2.91 | 28.53 | 2.87 | 1.27 | 4.66 | 0.57 |
|  | iHL | 32.33 | 7.48 | 26.60 | 10.37 | -5.73 | 17.72 | 0.92 |
| Onset 1 | iFL | 39.18 | 4.41 | 38.40 | 4.40 | -0.78 | 1.99 | 0.69 |
|  | iHL | 40.40 | 7.71 | 33.62 | 10.69 | -6.79 | 16.81 | 0.92 |
| Offset 2 | iFL | 76.34 | 0.85 | 75.84 | 0.86 | -0.51 | 0.67 | 0.32 |
|  | iHL | 75.19 | 1.11 | 73.43 | 1.12 | -1.77 | 2.35 | <0.0001 |
| Onset 2 | iFL | 97.09 | 6.77 | 86.30 | 6.80 | -10.8 | 11.12 | <0.0001 |
|  | iHL | 97.76 | 2.69 | 93.66 | 2.70 | -4.10 | 4.19 | 0.15 |
| Activity Duration | iFL | 72.11 | 3.38 | 65.36 | 3.39 | -6.75 | 9.36 | <0.0001 |
|  | iHL | 69.99 | 3.08 | 75.64 | 3.08 | 5.65 | 8.07 | <0.0001 |
| NLS Semitendinosus (% Stride) | | | | | | | | |
| Offset 1 | iFL | 18.58 | 1.53 | 23.16 | 1.51 | 4.58 | 24.65 | <0.0001 |
|  | iHL | 18.96 | 2.08 | 24.41 | 2.07 | 5.45 | 28.74 | <0.0001 |
| Onset 1 | iFL | 48.26 | 2.38 | 59.92 | 2.19 | 11.66 | 24.16 | <0.0001 |
|  | iHL | 52.10 | 4.02 | 52.67 | 4.03 | 0.57 | 1.09 | 0.64 |
| Offset 2 | iFL | 65.70 | 1.69 | 70.76 | 1.49 | 5.07 | 7.72 | <0.0001 |
|  | iHL | 64.06 | 4.82 | 65.74 | 4.83 | 1.68 | 2.62 | 0.19 |
| Onset 2 | iFL | 85.55 | 2.28 | 86.86 | 2.28 | 1.31 | 1.53 | 0.01 |
|  | iHL | 87.87 | 1.33 | 85.80 | 1.31 | -2.06 | 2.34 | 0.02 |
| Activity Duration | iFL | 38.15 | 4.42 | 44.28 | 4.39 | 6.13 | 16.07 | <0.0001 |
|  | iHL | 40.02 | 4.21 | 49.21 | 4.17 | 9.18 | 22.94 | <0.0001 |
| LS Semitendinosus (% Stride) | | | | | | | | |
| Offset 1 | iFL | 21.45 | 2.73 | 20.64 | 2.74 | -0.81 | 3.78 | 0.38 |
|  | iHL | 22.28 | 1.77 | 18.56 | 1.78 | -3.72 | 16.7 | <0.0001 |
| Onset 1 | iFL | 54.62 | 2.04 | 55.30 | 2.07 | 0.69 | 1.26 | 0.43 |
|  | iHL | 56.99 | 2.24 | 50.51 | 2.24 | -6.48 | 11.37 | <0.0001 |
| Offset 2 | iFL | 71.87 | 2.72 | 76.47 | 2.75 | 4.60 | 6.4 | <0.0001 |
|  | iHL | 73.89 | 2.53 | 66.25 | 2.53 | -7.64 | 10.34 | <0.0001 |
| Onset 2 | iFL | 90.20 | 1.65 | 88.81 | 1.66 | -1.40 | 1.55 | <0.0001 |
|  | iHL | 92.11 | 2.19 | 86.78 | 2.19 | -5.33 | 5.79 | <0.0001 |
| Activity Duration | iFL | 47.13 | 6.27 | 46.84 | 6.29 | -0.29 | 0.62 | 0.90 |
|  | iHL | 45.91 | 5.02 | 45.81 | 5.02 | -0.10 | 0.22 | 0.97 |
| NLS Triceps Brachii (% Stride) | | | | | | | | |
| Onset | iFL | 35.99 | 2.13 | 29.57 | 2.13 | -6.42 | 17.84 | <0.0001 |
|  | iHL | 37.67 | 2.69 | 32.04 | 2.68 | -5.64 | 14.97 | <0.0001 |
| Offset | iFL | 72.60 | 1.74 | 74.65 | 1.73 | 2.05 | 2.82 | <0.0001 |
|  | iHL | 71.80 | 1.14 | 77.88 | 1.14 | 6.09 | 8.48 | <0.0001 |
| Activity Duration | iFL | 39.46 | 3.44 | 47.11 | 3.43 | 7.65 | 19.39 | <0.0001 |
|  | iHL | 34.22 | 3.59 | 48.73 | 3.59 | 14.51 | 42.4 | <0.0001 |
| LS Triceps Brachii (% Stride) | | | | | | | | |
| Onset | iFL | 34.21 | 1.94 | 38.42 | 1.94 | 4.21 | 12.31 | <0.0001 |
|  | iHL | 36.60 | 2.93 | 36.60 | 2.93 | -0.01 | 0.03 | 0.99 |
| Offset | iFL | 73.26 | 0.99 | 73.00 | 0.99 | -0.27 | 0.37 | 0.68 |
|  | iHL | 76.30 | 0.77 | 72.24 | 0.77 | -4.07 | 5.33 | <0.0001 |
| Activity Duration | iFL | 43.69 | 2.85 | 40.97 | 2.85 | -2.71 | 6.20 | 0.03 |
|  | iHL | 41.06 | 3.09 | 37.68 | 3.09 | -3.38 | 8.23 | <0.0001 |
| NLS Latissimus Dorsi (% Stride) | | | | | | | | |
| Onset 1 | iFL | 11.25 | 1.20 | 10.43 | 1.22 | -0.81 | 7.20 | 0.11 |
|  | iHL | 10.96 | 1.03 | 10.63 | 1.02 | -0.32 | 2.92 | 0.58 |
| Offset 1 | iFL | 24.96 | 1.04 | 23.98 | 1.07 | -0.98 | 3.93 | 0.07 |
|  | iHL | 24.86 | 1.09 | 29.90 | 1.15 | 5.04 | 20.27 | <0.0001 |
| Onset 2 | iFL | 38.09 | 0.90 | 36.69 | 0.91 | -1.40 | 3.68 | <0.0001 |
|  | iHL | 37.82 | 1.27 | 42.44 | 1.32 | 4.62 | 12.22 | <0.0001 |
| Offset 2 | iFL | 77.42 | 2.02 | 77.16 | 2.02 | -0.26 | 0.34 | 0.69 |
|  | iHL | 76.91 | 0.70 | 82.78 | 0.70 | 5.87 | 7.63 | <0.0001 |
| Activity Duration | iFL | 52.39 | 3.51 | 52.47 | 3.51 | 0.08 | 0.15 | 0.97 |
|  | iHL | 52.57 | 1.58 | 63.09 | 1.57 | 10.53 | 20.03 | <0.0001 |
| LS Latissimus Dorsi (% Stride) | | | | | | | | |
| Onset 1 | iFL | 11.42 | 1.23 | 9.90 | 1.24 | -1.53 | 13.40 | <0.0001 |
|  | iHL | 12.02 | 1.53 | 9.62 | 1.53 | -2.40 | 19.97 | <0.0001 |
| Offset 1 | iFL | 25.95 | 1.41 | 24.66 | 1.42 | -1.29 | 4.97 | 0.02 |
|  | iHL | 26.87 | 1.41 | 22.55 | 1.41 | -4.32 | 16.08 | <0.0001 |
| Onset 2 | iFL | 41.24 | 2.00 | 39.24 | 2.00 | -2.00 | 4.85 | <0.0001 |
|  | iHL | 41.69 | 1.50 | 34.93 | 1.50 | -6.77 | 16.24 | <0.0001 |
| Offset 2 | iFL | 80.77 | 1.64 | 80.76 | 1.64 | -0.01 | 0.01 | 0.98 |
|  | iHL | 80.04 | 1.39 | 74.70 | 1.39 | -5.34 | 6.67 | <0.0001 |
| Activity Duration | iFL | 56.88 | 2.88 | 55.52 | 2.88 | -1.36 | 2.39 | 0.41 |
|  | iHL | 54.41 | 3.53 | 52.41 | 3.52 | -2.00 | 3.68 | 0.21 |

Table S4. Results from SPM analysis of sEMG data from a representative horse (Horse 1), presenting information on the data clusters that exceeded the critical thresholds for significance between baseline and induced forelimb lameness conditions. Data are presented for sEMG variables (muscles) from the lame side (LS), and non-lame side (NLS) limbs, where significant differences/data clusters were detected. The number of data clusters identified per kinematic variable (n clusters) are presented. P values, critical thresholds, and the beginning and end time points (as a percentage of stride) are presented for each data cluster.

| **Variable (Muscle)** | **Limb** | **n Clusters** | **Cluster Range**  **(% stride)** | | **p-value** | **Critical Threshold** |
| --- | --- | --- | --- | --- | --- | --- |
|  |  |  | **Start** | **End** |  |  |
| Latissimus dorsi | NLS | 1 | 65.38 | 66.11 | 0.02 | 4.44 |
| Triceps Brachii | LS | 2 | 16.74 | 17.79 | 0.01 | 4.68 |
|  |  |  | 20.72 | 21.71 | 0.01 |  |
|  | NLS | 5 | 18.97 | 22.23 | <0.0001 | 4.44 |
|  |  |  | 26.80 | 29.14 | <0.0001 |  |
|  |  |  | 43.09 | 46.00 | <0.0001 |  |
|  |  |  | 54.59 | 57.20 | <0.0001 |  |
|  |  |  | 69.06 | 70.36 | 0.01 |  |
| Superficial Gluteal | LS | 6 | 1.57 | 16.89 | <0.0001 | 4.73 |
|  |  |  | 17.07 | 33.74 | <0.0001 |  |
|  |  |  | 36.82 | 38.95 | <0.0001 |  |
|  |  |  | 64.20 | 70.63 | <0.0001 |  |
|  |  |  | 77.21 | 81.68 | <0.0001 |  |
|  |  |  | 90.19 | 91.40 | <0.0001 |  |
|  | NLS | 1 | 15.49 | 20.46 | <0.0001 | 4.73 |
| Biceps Femoris | LS | 6 | 0.05 | 2.45 | <0.0001 | 4.73 |
|  |  |  | 16.41 | 21.80 | <0.0001 |  |
|  |  |  | 40.21 | 45.48 | <0.0001 |  |
|  |  |  | 48.66 | 49.40 | 0.01 |  |
|  |  |  | 51.04 | 54.53 | <0.0001 |  |
|  |  |  | 99.85 | 100.00 | 0.02 |  |
|  | NLS | 6 | 36.53 | 38.33 | <0.0001 | 4.73 |
|  |  |  | 46.03 | 48.22 | <0.0001 |  |
|  |  |  | 53.67 | 54.30 | 0.01 |  |
|  |  |  | 62.72 | 63.16 | 0.02 |  |
|  |  |  | 69.37 | 70.54 | <0.0001 |  |
|  |  |  | 81.93 | 84.30 | <0.0001 |  |
| Semitendinosus | NLS | 3 | 14.99 | 15.18 | 0.02 | 4.73 |
|  |  |  | 71.20 | 73.59 | <0.0001 |  |
|  |  |  | 77.42 | 79.56 | <0.0001 |  |

Table S5. Results from SPM analysis of sEMG data from a representative horse (Horse 7), presenting information on the data clusters that exceeded the critical thresholds for significance between baseline and induced hindlimb lameness conditions. Data are presented for sEMG variables (muscles) from the lame side (LS), and non-lame side (NLS) limbs, where significant differences/data clusters were detected. The number of data clusters identified per kinematic variable (n clusters) are presented. P values, critical thresholds, and the beginning and end time points (as a percentage of stride) are presented for each data cluster.

| **Variable (Muscle)** | **Limb** | **n Clusters** | **Cluster Range**  **(% stride)** | | **p-value** | **Critical Threshold** |
| --- | --- | --- | --- | --- | --- | --- |
|  |  |  | **Start** | **End** |  |  |
| Latissimus Dorsi | LS | 7 | 14.91 | 15.18 | 0.03 | 4.55 |
|  |  |  | 25.12 | 29.82 | <0.0001 |  |
|  |  |  | 34.96 | 35.11 | 0.03 |  |
|  |  |  | 44.19 | 45.89 | <0.0001 |  |
|  |  |  | 54.93 | 55.10 | 0.03 |  |
|  |  |  | 58.31 | 60.33 | <0.0001 |  |
|  |  |  | 86.64 | 88.11 | <0.0001 |  |
|  | NLS | 2 | 23.38 | 24.49 | 0.01 | 4.61 |
|  |  |  | 59.98 | 61.15 | 0.01 |  |
| Triceps Brachii | LS | 3 | 37.48 | 40.08 | <0.0001 | 4.55 |
|  |  |  | 49.36 | 51.01 | <0.0001 |  |
|  |  |  | 53.80 | 56.48 | <0.0001 |  |
|  | NLS | 8 | 20.97 | 24.58 | <0.0001 | 4.61 |
|  |  |  | 33.75 | 35.09 | <0.0001 |  |
|  |  |  | 52.45 | 54.83 | <0.0001 |  |
|  |  |  | 59.68 | 62.31 | <0.0001 |  |
|  |  |  | 69.36 | 74.48 | <0.0001 |  |
|  |  |  | 76.57 | 77.34 | 0.02 |  |
|  |  |  | 85.96 | 87.01 | 0.01 |  |
|  |  |  | 95.67 | 96.68 | 0.01 |  |
| Superficial Gluteal | LS | 6 | 0.00 | 2.23 | <0.0001 | 4.97 |
|  |  |  | 12.18 | 13.14 | 0.01 |  |
|  |  |  | 34.69 | 38.61 | <0.0001 |  |
|  |  |  | 48.20 | 51.32 | <0.0001 |  |
|  |  |  | 83.69 | 85.95 | <0.0001 |  |
|  |  |  | 97.31 | 100.00 | <0.0001 |  |
|  | NLS | 6 | 3.60 | 4.10 | 0.02 | 4.73 |
|  |  |  | 10.07 | 11.95 | <0.0001 |  |
|  |  |  | 35.94 | 38.02 | <0.0001 |  |
|  |  |  | 62.96 | 63.04 | 0.02 |  |
|  |  |  | 70.09 | 79.30 | <0.0001 |  |
|  |  |  | 89.34 | 94.47 | <0.0001 |  |
| Biceps Femoris | LS | 10 | 0.00 | 2.37 | <0.0001 | 4.97 |
|  |  |  | 7.61 | 12.17 | <0.0001 |  |
|  |  |  | 17.95 | 22.61 | <0.0001 |  |
|  |  |  | 40.85 | 43.40 | <0.0001 |  |
|  |  |  | 46.48 | 48.25 | <0.0001 |  |
|  |  |  | 56.01 | 61.74 | <0.0001 |  |
|  |  |  | 64.77 | 68.11 | <0.0001 |  |
|  |  |  | 69.99 | 71.74 | <0.0001 |  |
|  |  |  | 77.53 | 84.85 | <0.0001 |  |
|  |  |  | 91.95 | 100.00 | <0.0001 |  |
|  | NLS | 5 | 10.43 | 15.28 | <0.0001 | 4.73 |
|  |  |  | 21.37 | 22.20 | 0.01 |  |
|  |  |  | 31.64 | 35.62 | <0.0001 |  |
|  |  |  | 37.54 | 65.13 | <0.0001 |  |
|  |  |  | 70.72 | 83.29 | <0.0001 |  |
| Semitendinosus | LS | 5 | 51.66 | 54.01 | <0.0001 | 4.97 |
|  |  |  | 58.36 | 64.33 | <0.0001 |  |
|  |  |  | 66.87 | 68.69 | <0.0001 |  |
|  |  |  | 81.35 | 85.97 | <0.0001 |  |
|  |  |  | 89.61 | 98.87 | <0.0001 |  |
|  | NLS | 7 | 0.00 | 1.93 | <0.0001 | 4.73 |
|  |  |  | 3.83 | 5.96 | <0.0001 |  |
|  |  |  | 27.73 | 31.45 | <0.0001 |  |
|  |  |  | 58.60 | 66.04 | <0.0001 |  |
|  |  |  | 69.39 | 72.05 | <0.0001 |  |
|  |  |  | 90.28 | 91.83 | <0.0001 |  |
|  |  |  | 98.58 | 100.00 | <0.0001 |  |
